# Bioinspired Nanoplatform Potentiates Sonodynamic Immunotherapy by Remodeling the Antioxidant Tumor Microenvironment and Activating STING pathway

**DOI:** 10.7150/thno.129765

**Published:** 2026-04-16

**Authors:** Yuanyuan Zhang, Wenxiang Zhu, Kaimin Li, Yun Xie, Zhichao Deng, Yuanyuan Zhu, Bowen Gao, Chenxi Xu, Junlong Fu, Mingzhen Zhang, Xiaoliang Zheng, Haifeng Zhang

**Affiliations:** 1 The Clinical Laboratory, Northwest Women's and Children's Hospital, Xi'an, Shaanxi, 710061, China.; 2 Nanozyme Laboratory in Zhongyuan, Henan Academy of Innovations in Medical Science, Zhengzhou, Henan, 451163, China.; 3 School of Biological and Medical Engineering, Beijing University of Aeronautics and Astronautics, Beijing, 100191, China.; 4 School of Basic Medical Sciences, Xi'an Jiaotong University, Xi'an, Shaanxi, 710061, China.; 5 School of Laboratory Medicine and Bioengineering, Hangzhou Medical College, Hangzhou, Zhejiang, 310053, China.; 6 Department of Pathology, School of Basic Medical Sciences, Xi'an Jiaotong University, Xi'an, Shaanxi, 710061, China.; 7 Institute of Genetics and Developmental Biology, Translational Medicine Institute, Xi'an Jiaotong University, Xi'an, Shaanxi, 710061, China.

**Keywords:** Sonodynamic therapy, Immunotherapy, Mn_3_O_4_ nanoparticles, Ag_2_S quantum dots, colon cancer

## Abstract

**Background:**

Sonodynamic therapy (SDT) has emerged as a promising modality for treating deep-seated tumors. It has been demonstrated that SDT effectively induces immunogenic cell death (ICD), thereby initiating a systemic anti-tumor immune response — a process known as sonodynamic immunotherapy. However, its efficacy is severely limited by the hypoxic tumor microenvironment (TME) and elevated glutathione (GSH) levels, which together scavenge reactive oxygen species (ROS) and create a potent antioxidant barrier.

**Methods:**

Ultra-small Mn_3_O_4_ nanoparticles with multi-enzyme mimicking activity were synthesized, and co-encapsulated with the sound sensitizer Ag_2_S quantum dot (Ag_2_S QD) in cell membrane hybrid liposomes to construct a biomimetic nanoplatform (Mn_3_O_4_/QD@LM). The Catalase and glutathione peroxidase activities of Mn_3_O_4_/QD@LM were evaluated. Its antitumor efficacy *in vitro* was evaluated by measuring ROS levels, mitochondrial membrane potential staining, live/dead cell staining, and apoptosis analysis. By recording tumor growth and performing histological and immunohistochemical examinations, its antitumor effects *in vivo* were investigated in a mouse model of colon cancer. Flow cytometry analysis was used to analyze the tumor immune microenvironment.

**Results:**

Mn_3_O_4_/QD@LM functioned as a “ROS amplifier” by exhibiting catalase-like and glutathione peroxidase-like activities, which alleviated tumor hypoxia and depleted GSH, thereby markedly enhancing SDT efficacy. Moreover, released Mn^2+^ ions generated highly cytotoxic hydroxyl radicals via a Fenton-like reaction, further augmenting tumor cell killing. *In vitro* experiments confirmed that Mn_3_O_4_/QD@LM effectively induced ICD and activated the STING pathway. Benefiting from homologous targeting, the nanoplatform achieved efficient accumulation in tumor tissue *in vivo*. Upon ultrasound activation, Mn_3_O_4_/QD@LM significantly inhibited tumor growth both *in vitro* and *in vivo*. Notably, it remodeled the tumor immune microenvironment by promoting CD8⁺ T cell infiltration, enhancing the secretion of IFN-γ and TNF-α, and reducing the populations of regulatory T cells and myeloid-derived suppressor cells.

**Conclusions:**

Mn_3_O_4_/QD@LM confirms the synergistic role of multi-enzyme activities and STING pathway activation in potentiating sonodynamic immunotherapy, and provides an innovative strategy to overcome TME-mediated therapy resistance.

## Introduction

Sonodynamic therapy (SDT) is a non-invasive tumor treatment that uses ultrasound to activate sonosensitizers and generate reactive oxygen species (ROS) 1. These ROS induce oxidative damage to tumor cellular components, ultimately leading to cell death 2. Emerging evidence suggests that this cytotoxic effect can trigger immunogenic cell death (ICD). This process is characterized by the release of damage-associated molecular patterns (DAMPs), including calreticulin (CRT) and high-mobility group box 1 (HMGB1) protein, which bind to dendritic cells (DCs) receptors, promoting DCs maturation and antigen presentation. This cascade ultimately initiates an effective anti-tumor immune response 3, 4.

However, the efficacy of SDT and its immune effects are severely limited by the tumor antioxidant microenvironment (TME). One major limitation is the imbalance between oxygen supply and demand within tumor tissue. Over 50% of solid tumors contain severely hypoxic regions (partial oxygen pressure <10 mmHg) 5. Hypoxia not only reduces the production of O_2_-dependent ROS but also promotes immunosuppression by upregulating immune checkpoints such as PD-L1 6. Another limiting factor is the antioxidant defense barrier established by tumor cells through the overexpression of glutathione (GSH). The thiol group in GSH can directly neutralize ROS and significantly reduce the oxidative damage efficiency of SDT 7, 8. The interaction of hypoxia and antioxidant systems can also promote tumor metastasis, drug resistance, and immune evasion 9. Furthermore, even if SDT successfully induces ICD, it often fails to fully stimulate DC maturation, limiting subsequent antigen presentation and T cell activation. Therefore, the key to improving the efficacy of sonodynamic immunotherapy is to simultaneously break the physicochemical barriers of the TME and enhance the immune activation effect of SDT.

Transition metal manganese has attracted much attention due to its multivalent state, adjustable redox activity, and good biocompatibility. Engineered manganese oxide nanoparticles (MnOx NPs) can modulate TME by altering redox states and promoting ROS production 10. First, MnOx NPs exhibit catalase (CAT)-like activity in the weakly acidic TME, breaking down endogenous H_2_O_2_ into O_2_ 11. Second, high-valence MnOx (e.g., Mn^3+^/Mn^4+^) can effectively consume GSH through redox reactions (i.e., glutathione peroxidase (GPx)-like activity), disrupting the antioxidant barrier of tumors 12. Most importantly, the Mn^2+^ ions generated from these redox reactions can trigger Fenton-like reactions, converting the less cytotoxic H_2_O_2_ into highly oxidative hydroxyl radicals (∙OH) 13. This generates a synergistic effect with the ROS produced by SDT, significantly elevating oxidative stress levels within tumor cells.

Beyond its role in redox modulation, Mn^2+^ acts as a key immunomodulator that can activate the stimulator of interferon genes (STING) signaling pathway 14. STING activation promotes type I interferon secretion, DCs and macrophage maturation, and enhances CD8⁺ T cell priming and memory responses 15-19. Therefore, combining SDT with the immunomodulatory effect of Mn^2+^ creates a dual synergy. On one hand, SDT induces ICD to initiate immune activation. On the other hand, Mn^2+^ activates the STING pathway to further boost immunity. This dual synergy can strongly amplify anti-tumor immune responses.

Mn_3_O_4_ nanoparticles (Mn_3_O_4_ NPs) are relatively small in size and have excellent enzyme catalytic activity 20, 21. Therefore, we prioritized Mn_3_O_4_ NPs that are smaller in size and have stronger enzyme catalytic activity in the SDT process to achieve oxygen supply and GSH consumption. Meanwhile, sulfide quantum dots (Ag_2_S QDs) are emerging as potent sonosensitizers, characterized by their excellent biocompatibility, prolonged half-life in blood circulation, and NIR-II imaging performance 22, 23. These characteristics establish Ag_2_S QDs as a valuable candidate with considerable potential for translational applications in SDT. Based on the above advantages, this study fabricated a multifunctional nanotherapeutic platform (Mn_3_O_4_/QD@LM). Using cancer membrane-hybridized liposomes as the carrier, the platform co-delivers Ag_2_S QDs as sonosensitizers and ultra-small Mn_3_O_4_ NPs. This system integrates homologous targeting, TME remodeling, and dual immune activation functions to achieve synergistic enhancement of SDT and anti-tumor immunity.

**Figure 1:**
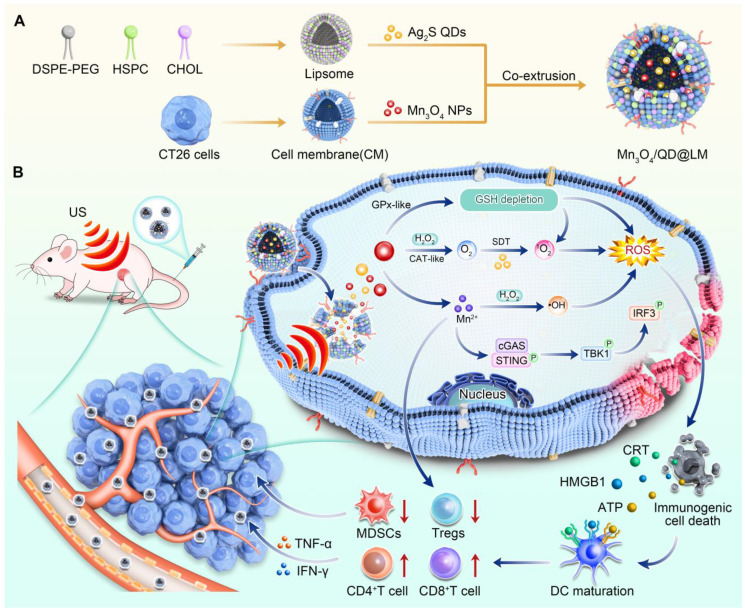
** Schematic diagram of Mn_3_O_4_/QD@LM potentiates sonodynamic immunotherapy by remodeling antioxidant tumor microenvironment and activating STING pathway. (A)** The synthetic route of Mn_3_O_4_/QD@LM. **(B)** The mechanisms of sonodynamic immunotherapy mediated by Mn_3_O_4_/QD@LM. Following intravenous injection, Mn_3_O_4_/QD@LM targets the tumor through homologous cell membrane-mediated homing. In the tumor microenvironment, Mn_3_O_4_/QD@LM exhibits CAT-like and GPx-like activities, depleting GSH and generating ·OH. Upon exposure to US irradiation, Mn_3_O_4_/QD@LM induces immunogenic cell death. Furthermore, the released Mn²⁺ activates the cGAS-STING pathway. Consequently, Mn_3_O_4_/QD@LM significantly increased the number of CD8⁺ T cells and elevated the levels of cytokines such as IFN-γ and TNF-α, while reducing the infiltration of Tregs and MDSCs, leading to effective suppression of tumor growth.

Specifically, cancer cell membranes confer homologous targeting capabilities on nanoplatforms, enabling their effective accumulation in tumor tissue. At the tumor site, Mn_3_O_4_ NPs break down endogenous H_2_O_2_ into O_2_ via CAT-like activity, effectively relieving tumor hypoxia. At the same time, Mn_3_O_4_ NPs deplete GSH through Gpx-like activity, breaking the antioxidant barrier. These effects create favorable conditions for Ag_2_S QDs to generate abundant ROS under ultrasound. Increased ROS production can induce stronger ICD, which can increase the release of DAMPs and TAAs. Concurrently, Mn^2+^ drives Fenton-like reactions and activates the STING pathway, promoting DC maturation and pro-inflammatory cytokine secretion. In a mouse CT26 tumor model, this system significantly increased tumor-infiltrating CD8⁺ T cells and cytokine levels (IFN-γ, TNF-α), while reducing immunosuppressive regulatory T cells (Tregs) and myeloid-derived suppressor cells (MDSCs) populations, effectively suppressing tumor growth **(Figure 1)**.

In summary, the Mn_3_O_4_/QD@LM system constructed in this study possesses both multienzyme-mimetic activities and immunomodulatory functions. It not only overcomes the core bottlenecks of tumor hypoxia and antioxidant barriers in SDT, but also achieves dual synergy between ICD-mediated immune activation and Mn^2+^-triggered STING pathway activation. This strategy synchronously enhances the sonodynamic therapeutic effect and anti-tumor immune response. Thus, this work presents a novel strategy for enhancing the efficacy of sonodynamic immunotherapy in colon cancer.

## Methods

### Preparation of Ag_2_S QDs, Mn_3_O_4_ NPS, and Mn_3_O_4_/QD@LM

Ag_2_S QDs were synthesized according to our previous method 24. Synthesis of ultrasmall Mn_3_O_4_ NPs was performed according to a literature method with modifications 20. Specifically, 300 mg manganese (II) acetylacetonate and 9.63 mL oleamide were added to a 100 mL three-neck flask. The system was purged with N_2_ for 30 min to remove oxygen, followed by heating to 150 °C at 5 °C/min under N_2_ atmosphere and maintaining for 9 h. After natural cooling to room temperature, excess ethanol/cyclohexane (3:1 v/v) was added dropwise to wash the solution three times, removing unreacted precursors and surfactants. The precipitate was collected by high-speed centrifugation (12,000 rpm, 15 min). Finally, the obtained precipitate was re-dispersed in hexane for further use.

Synthesis of hybrid liposome Mn_3_O_4_/QD@LM: (1) Lipid film preparation: Hydrogenated soy phosphatidylcholine, cholesterol, and DSPE-PEG2000 (1:1:0.13 molar ratio) were dissolved in 3 mL dichloromethane. Then, 5 mg Ag_2_S QDs and 1 mg Mn_3_O_4_ nanoparticles were added. The solution underwent rotary evaporation at 50 °C to form a thin film. The flask was vacuum-dried for 12 h to remove residual solvents. (2) Membrane hybridization and hydration: The lipid film was mixed with pre-extracted CT26 cell membranes [4:1 (w/w)] in 4 mL PBS. Membrane fusion was facilitated by oscillatory hydration (300 rpm, 37 °C, 30 min). (3) The hydrated system was dispersed by probe sonication (ice bath, 2 min) and sequentially extruded 20 times through 400 nm and 200 nm polycarbonate membranes using an Avanti mini-extruder. Unencapsulated Ag_2_S QDs and Mn_3_O_4_ NPs were removed by centrifugation (3000 rpm, 10 min). The final product (Mn_3_O_4_/QD@LM) was stored at 4 °C under light-protected conditions. Mn_3_O_4_/QD@Lip was prepared identically except for the omission of CT26 cell membranes.

### Determination of catalase-like activity of Mn_3_O_4_/QD@LM

The catalase-like activity of Mn_3_O_4_/QD@LM was detected through dissolved oxygen detection experiments and H_2_O_2_ consumption experiments.

(1) Dissolved oxygen detection assay. After calibrating the dissolved oxygen meter, the automatic recording mode was configured in the software with a recording interval of 1 min. Then, 10 mL of 1 mM H_2_O_2_ solution was measured and transferred into a clean scintillation vial. Different concentrations of Mn_3_O_4_/QD@LM were added to the vial, and continuous monitoring and data recording were initiated. The oxygen production from 1 mM H_2_O_2_ solution alone served as the control group. All experiments were repeated three times to guarantee the reliability of the data.

(2) H_2_O_2_ consumption assay. The solution of Mn_3_O_4_/QD@LM was serially diluted to target concentrations. Then, 1 mL of each diluted solution was mixed with 10 μM H_2_O_2_-specific fluorescent probe ROS Green^TM^. Afterward, 10 μL of 10 mM H_2_O_2_ solution was added to each mixture and thoroughly mixed. The reaction tubes were incubated in the dark with shaking for 4 h. After incubation, 100 μL of reaction solution was taken from each sample, and the fluorescence intensity was measured using a multifunctional microplate reader (ex: 488 nm; em: 515 nm). All experiments were repeated three times to guarantee the reliability of the data.

### Determination of glutathione peroxidase-like activity of Mn_3_O_4_/QD@LM

The glutathione peroxidase-like activity of Mn_3_O_4_/QD@LM was quantitatively analyzed using the DTNB colorimetric assay. DTNB specifically reacts with free thiol groups to produce a yellow-colored product, whose absorbance is directly proportional to the concentration of GSH. Different concentrations of Mn_3_O_4_/QD@LM were mixed with 200 μM GSH in a total volume of 1 mL. The mixtures were incubated in the dark at 37 °C with shaking for 3 h. After the reaction, the mixtures were centrifuged at 12000 g for 5 min to remove the nanoparticle precipitates. Upon collection, 800 μL of the supernatant was mixed with a DTNB solution (200 μM) and incubated at room temperature in the dark for 1 h. A 200 μL aliquot of the mixture was transferred to a 96-well plate, and its absorption spectrum was scanned from 400 to 600 nm using a UV-vis spectrophotometer. At the same time, the absorbance of the remaining solution was measured at a wavelength of 412 nm to calculate the remaining GSH content.

### Evaluation of hydroxyl radical generation by Mn_3_O_4_/QD@LM

The ∙OH generation capability of Mn_3_O_4_/QD@LM was assessed using the methylene blue (MB) decolorization assay and electron spin resonance (ESR) spectroscopy.

(1) Methylene Blue decolorization assay. A reaction mixture containing 40 μg/mL Mn_3_O_4_/QD@LM nanoparticles and 4 mM GSH was prepared in water. The mixture was incubated in the dark at 37 °C with shaking for 1 h. After reaction, the solution was centrifuged at 12000 g for 5 min to completely remove unreacted nanoparticles. Then, 800 μL of the supernatant was mixed with the MB working solution (10 μg/mL) and H_2_O_2_ (8 mM). Control groups included MB alone and MB with H_2_O_2_. The final mixtures were incubated again at 37 °C in the dark for 1 h, after which the absorption spectra of MB were recorded over the 500-800 nm range using a UV-Vis spectrophotometer.

(2) ESR detection of ∙OH. Different concentration gradients of the nanoparticles were mixed with 4 mM GSH in a total volume of 1 mL, followed by incubation in the dark at 37 °C for 12 h. After reaction, the mixture was centrifuged at 2000 g for 15 min to remove unreacted nanoparticles. Then, 800 μL of the supernatant was combined with 10 μL of DMPO solution, and 10 μL of H_2_O_2_ was added to achieve a final concentration of 8 mM. The solution was vortexed thoroughly. Control groups included DMPO alone and DMPO with H_2_O_2_. All reaction systems were incubated in the dark at room temperature for 5 min, and then the DMPO-OH adduct signals were detected using an ESR spectrometer.

### Evaluation of singlet oxygen generation by Mn_3_O_4_/QD@LM

The singlet oxygen (^1^O_2_) generation capacity of Mn_3_O_4_/QD@LM was evaluated using the singlet oxygen sensor green (SOSG) fluorescent probe and ESR spectroscopy.

(1) SOSG fluorescence probe detection of ^1^O_2_. 1 mL solution of Mn_3_O_4_/QD@LM (50 μg/mL) was mixed with 10 μL of SOSG stock solution (5 μM). The mixture was then transferred to a 6-well plate and subjected to ultrasonication (1.5 W/cm^2^) for different durations. Immediately after ultrasonication, the fluorescence of the samples was measured using a fluorescence spectrophotometer (ex: 505 nm; emission scanning range: 510 - 600 nm).

(2) ESR detection of ^1^O_2_. 100 mM TEMP was added to different experimental groups: TEMP + Mn_3_O_4_/QD@LM, TEMP + Mn_3_O_4_/QD@LM + H_2_O_2_, TEMP + Mn_3_O_4_/QD@LM + US, TEMP + Mn_3_O_4_/QD@LM + H_2_O_2_ + US. (Mn_3_O_4_/QD@LM: 50 μg/mL; H_2_O_2_: 1 mM). The ultrasonication-treated groups were exposed to ultrasound irradiation (1.0 W/cm^2^, 3 min) using an ultrasonic instrument. After ultrasound irradiation, the reaction solution was immediately transferred to a quartz capillary for ESR measurement.

### Cells and animals

The murine colon cancer cell line CT26 and the human colon cancer cell line Caco-2 were obtained from ATCC. Cultures were maintained in RPMI 1640 complete medium at 37 °C in a humidified atmosphere containing 5% CO_2_. Cell authenticity was verified by morphological examination and PCR analysis, confirming the absence of interspecies cross-contamination.

Female Balb/c mice (6-8 weeks old) were purchased from GemPharmatech Co., Ltd. (Jiangsu, China). Mice were housed under SPF conditions. The environment featured a controlled temperature and humidity, along with a 12-h light/dark cycle. All experimental procedures involving animals followed national guidelines. Ethical approval was granted by the Animal Ethics Committee of Xi'an Jiaotong University.

### *In vitro* anticancer efficacy of Mn_3_O_4_/QD@LM

CT26 cells were seeded in 12-well plates (1×10^5^ cells per well) and cultured overnight for adherence. For the experimental groups, cells were treated with various nanoparticles (Mn_3_O_4_: 5 μg/mL; Ag_2_S QDs: 25 μg/mL). After 8 h of incubation, the cells were washed three times with PBS before adding fresh medium. Cells in the ultrasound groups were then subjected to US treatment (1.0 W/cm^2^, 3 min). Subsequently, different staining procedures were performed according to the steps described below, along with corresponding analyses.

(1) Calcein-AM/PI staining. After a 6-h incubation period following US treatment, the cells were stained with 2 μM Calcein-AM and 1 μg/mL PI in PBS for 30 min in the dark. The staining solution was then removed, and the cells were washed with PBS. Fluorescence microscopy was subsequently performed, capturing images from randomly selected fields per well using a consistent exposure time.

(2) DCFH-DA staining. Immediately after US treatment, cells were incubated with serum-free medium containing 10 μM DCFH-DA probe for 30 min at 37 °C in the dark. After washing with PBS, images were acquired using a fluorescence microscope with a consistent exposure time from randomly selected fields in each well.

(3) JC-1 staining. After US treatment, continue to culture the cells for 3 h. Then, 5 μg/mL JC-1 staining solution was added and incubated for 20 min at 37 °C. Changes in mitochondrial membrane potential were observed under fluorescence microscope. For flow cytometry analysis, cells are collected into centrifuge tubes after incubation and staining. After staining, the cells were washed twice with washing buffer and tested on the machine.

(4) Annexin V-FITC/PI staining. After US treatment, cells were cultured for another 6 h. Digest the cells by trypsin without EDTA, centrifuge at 1200 rpm for 5 min to pellet the cells. After two washes of cells with pre-chilled PBS, the cells were resuspended in 100 μL of staining buffer at a concentration of approximately 1×10^6^ cells/mL. After adding 5 μL of Annexin V-FITC and 5 μL of PI, the mixture was gently vortexed and incubated for 15 min at room temperature in the dark. Stained cells were then immediately analyzed using a flow cytometer.

### Mn_3_O_4_/QD@LM biosafety evaluation

Balb/c mice were randomly divided into three groups (n = 5). Mice in the experimental group received Mn_3_O_4_/QD@LM tail vein injection every three days for a total of 3 doses. The control group was given the same volume of saline. Mice were euthanized on day 15 or 30 after the first injection. After sacrificing mice, their blood is collected for routine blood and biochemical tests. At the same time, major organs are examined histopathologically by H&E staining.

### *In vivo* anticancer efficacy of Mn_3_O_4_/QD@LM

To establish colon cancer models, 4-6-week-old Balb/c mice received a subcutaneous inoculation of 1×10^6^ colon cancer cells. When tumors attained a volume of 50-80 mm^3^, the mice were randomly separated into six groups: G1: Control, G2: US, G3: Mn_3_O_4_@LM, G4: QD@LM + US, G5: Mn_3_O_4_/QD@LM, G6: Mn_3_O_4_/QD@LM + US. Different nanoparticles were administered via tail vein injection on days 0, 4, 8, and 12. For groups receiving ultrasound treatment (G2, G4, G6), irradiation (1.5 W/cm^2^, 3 min) was performed 24 h post-injection. Mouse body weight and tumor volume were recorded every two days. Tumor volume (V) was estimated using the formula: V = (width² × length) × 0.5. Upon completion of the study, mice were euthanized; tumors and vital organs were harvested. Histological analysis of tumor tissues was performed using H&E, TUNEL, and Ki67 staining to assess tumor cell proliferation, apoptosis, and pathological changes.

### Immune cell subpopulation analysis

Tumor tissues from each treatment group were processed into single-cell suspensions for flow cytometry. Chopped tumor fragments were digested in dissociation buffer containing 100 μg/mL DNAse I and 1 mg/mL Collagenase IV, with continuous shaking for 60 min at 37 °C. The digested mixture was filtered through a cell strainer. Cells were washed twice with ice-cold PBS via centrifugation. Next, cells were resuspended in staining buffer. The following fluorescent conjugated antibodies were added to the cell suspension to stain the surface markers: APC/Cyanine7-anti-CD45, PE/Cyanine7-anti-CD8a, FITC-anti-CD11b, PerCP/Cyanine5.5-anti-CD11c, Pacific Blue™-anti-CD4, PE-anti-Ly6G, PE/Cyanine7-anti-Ly6C, Pacific Blue™-anti-MHCII. After surface staining, cells were washed twice with ice-cold PBS. Cells were then fixed, permeabilized, and stained for intracellular markers: PE-anti-TNF-α, APC-anti-IFN-γ, PE-anti-Foxp3.

Stained cells were analyzed using a flow cytometer. Acquired data were processed with FlowJo software to quantify the proportions of these immune cell subsets: mature DCs (CD45⁺CD11c⁺MHC II⁺), CD8⁺ T cells (CD45⁺CD8⁺), functional CD8⁺ T cells (CD45⁺CD8⁺IFN-γ⁺/TNF-α⁺), functional CD4⁺ T cells (CD45⁺CD4⁺IFN-γ⁺/TNF-α⁺), Tregs (CD45⁺CD4⁺Foxp3⁺), and MDSCs (CD45⁺CD11b⁺Ly6G ⁺).

### Ultrasound parameters

The experiment employed an ultrasound therapy device (Model WED-100, manufactured by China's Weierde Company) as the ultrasound trigger source. This device operates using pulsed wave irradiation and is configured with the following parameters: an output power of 1.0 W/cm^2^ for *in vitro* studies and 1.5 W/cm^2^ for *in vivo* applications, a working frequency of 1.0 MHz, a duty cycle set at 50%, an effective irradiation area of 2.0 cm², and a pulsed repetition frequency of 10 ms. Throughout the procedure, the ultrasound probe was positioned to ensure consistent contact and irradiation coverage.

### Statistical analysis

Statistical analysis was performed using GraphPad Prism software. Data are expressed as mean ± SD from at least three independent experiments. Student's t-test was used for two-group comparisons, and one-way analysis of variance (ANOVA) with Tukey's post-hoc test was applied for multiple group comparisons. Statistical significance is denoted by *p < 0.05, **p < 0.01, and ***p < 0.001.

## Results and Discussion

### Synthesis and characterization of Mn_3_O_4_/QD@LM

**Figure 2:**
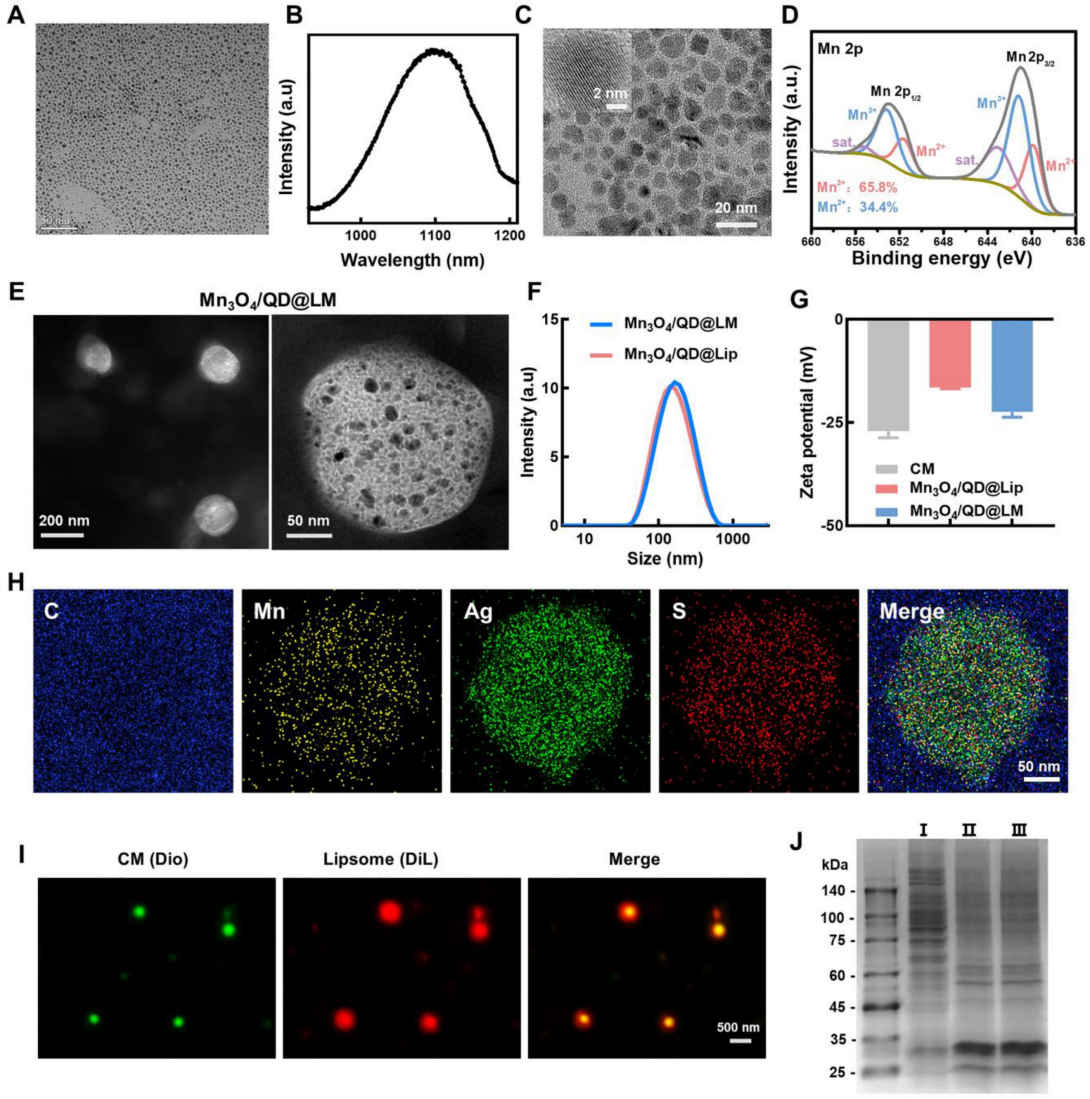
** Synthesis and characterization of Mn_3_O_4_/QD@LM. (A)** TEM images of Ag_2_S QDs. **(B)** Fluorescence spectra of Ag_2_S QDs. **(C)** TEM images of Mn_3_O_4_ NPs. **(D)** XPS spectra for the Mn 2p peak of Mn_3_O_4_ NPs. **(E)** TEM images of Mn_3_O_4_/QD@LM. **(F)** Size distribution of Mn_3_O_4_/QD@Lip and Mn_3_O_4_/QD@LM. **(G)** Zeta potential of CM, and Mn_3_O_4_/QD@Lip and Mn_3_O_4_/QD@LM. **(H)** TEM elemental mapping images of Mn_3_O_4_/QD@LM. **(I)** Fluorescence staining images of Dio-labeled CM, DiL-labeled liposome, and hybridization products. **(J)** SDS-PAGE image. I: cell lysate, II: cancer cell membrane, III: Mn_3_O_4_/QD@LM.

First, oil-dispersible Ag_2_S QDs were synthesized separately via a high-temperature pyrolysis method. Transmission electron microscopy (TEM) characterization shows that the QDs possess uniform size, good dispersity, and a diameter of approximately 4-5 nm (**Figure 2A**). The fluorescence spectrum in **Figure 2B** shows an emission peak at 1100 nm under 808 nm excitation. Next, the oil-soluble ultrasmall Mn_3_O_4_ NPs were prepared by a solution-phase method. TEM results show that the synthesized Mn_3_O_4_ NPs are monodisperse and spherical. High-resolution TEM reveals distinct lattice fringes, confirming their crystalline structure (**Figure 2C**). Size distribution analysis of 100 particles using Image J software yielded an average diameter of 8.1 ± 0.5 nm. The surface chemistry of the Mn_3_O_4_ NPs was further characterized by X-ray photoelectron spectroscopy (XPS). The XPS survey scan (**Figure S1A**) detected characteristic peaks at binding energies of 284.75 eV, 399.73 eV, 531.65 eV, and 641.08 eV, corresponding to C 1s, N 1s, O 1s, and Mn 2p, respectively, confirming that the surface chemical composition of the NPs is consistent with expectations. The Mn 3s orbital exhibits a typical doublet structure due to 3s-3d electron exchange interaction, with the main peak located at 82.7 eV and the satellite peak at 88.6 eV (**Figure S1B**). As shown in **Figure 2D**, the Mn 2p XPS spectrum of Mn_3_O_4_ shows typical spin-orbit splitting with Mn 2p_3/2_ and Mn 2p_1/2_ peaks at 641.1 eV and 653.0 eV, respectively. Meanwhile, distinct shake-up satellite peaks are observed on the high-binding-energy side of the main peaks, which are the characteristic signature of the Mn_3_O_4_ phase. Peak deconvolution reveals Mn^2+^ (34.4%) and Mn^3+^ (65.8%), with a ratio consistent with the 1:2 stoichiometry of Mn_3_O_4_ reported in the literature 25.

To develop a self-oxygenating nano-delivery system, liposomes (Mn_3_O_4_/QD@Lip) co-loaded with Mn_3_O_4_ NPs and Ag_2_S QDs were synthesized using hydrogenated soybean phosphatidylcholine, cholesterol, and DSPE-PEG2000. Subsequently, to enhance targeting capability, cancer cell membranes (CM) were incorporated into the liposomes. This successfully yielded biomimetic hybrid liposomes (Mn_3_O_4_/QD@LM). TEM images revealed that the hybrid liposomes possess a spherical morphology. Higher-magnification images further showed the interior to be uniformly and densely loaded with numerous ultrasmall Mn_3_O_4_ NPs and Ag_2_S QDs (**Figure 2E**). Dynamic light scattering (DLS) was then used to analyze the hydrodynamic size and zeta potential of Mn_3_O_4_/QD@Lip and Mn_3_O_4_/QD@LM. Size analysis showed similar dimensions: Mn_3_O_4_/QD@Lip at 143.6 ± 2.5 nm and Mn_3_O_4_/QD@LM at 148.7 ± 1.3 nm (**Figure 2F**). Zeta potential analysis showed a zeta value of about -28 mV for the isolated CM, a zeta value of about -16.1 mV for Mn_3_O_4_/QD@Lip, and a zeta value of -23.1 mV for Mn_3_O_4_/QD@LM (**Figure 2G**). This shift in zeta potential confirms the successful integration of cell membrane components into liposomes. At the same time, the element mapping image shows that the Mn, Ag, and S elements exhibit highly overlapping spatial distributions. All elements were evenly distributed, and there was no significant phase separation, indicating the structural integrity and good dispersion of this nanosystem (**Figure 2H**). To further verify the incorporation of the membrane, fluorescence imaging characterization was performed. The extracted cell membranes were labeled with DiO dye (green fluorescence) and liposomes with DiL dye (red fluorescence). The fluorescence images in **Figure 2I** show that the red and green signals in the synthesized liposomes are colocalized. At the same time, SDS-PAGE analysis showed that the protein bands of Mn_3_O_4_/QD@LM were similar to those of purified cell membranes, confirming that the cell membrane was successfully incorporated into liposomes (**Figure 2J**). This result further confirms the successful construction of biomimetic hybrid liposomes.

### Mn_3_O_4_/QD@LM exhibits enzyme-like activity and enhances sonodynamic therapy

**Figure 3:**
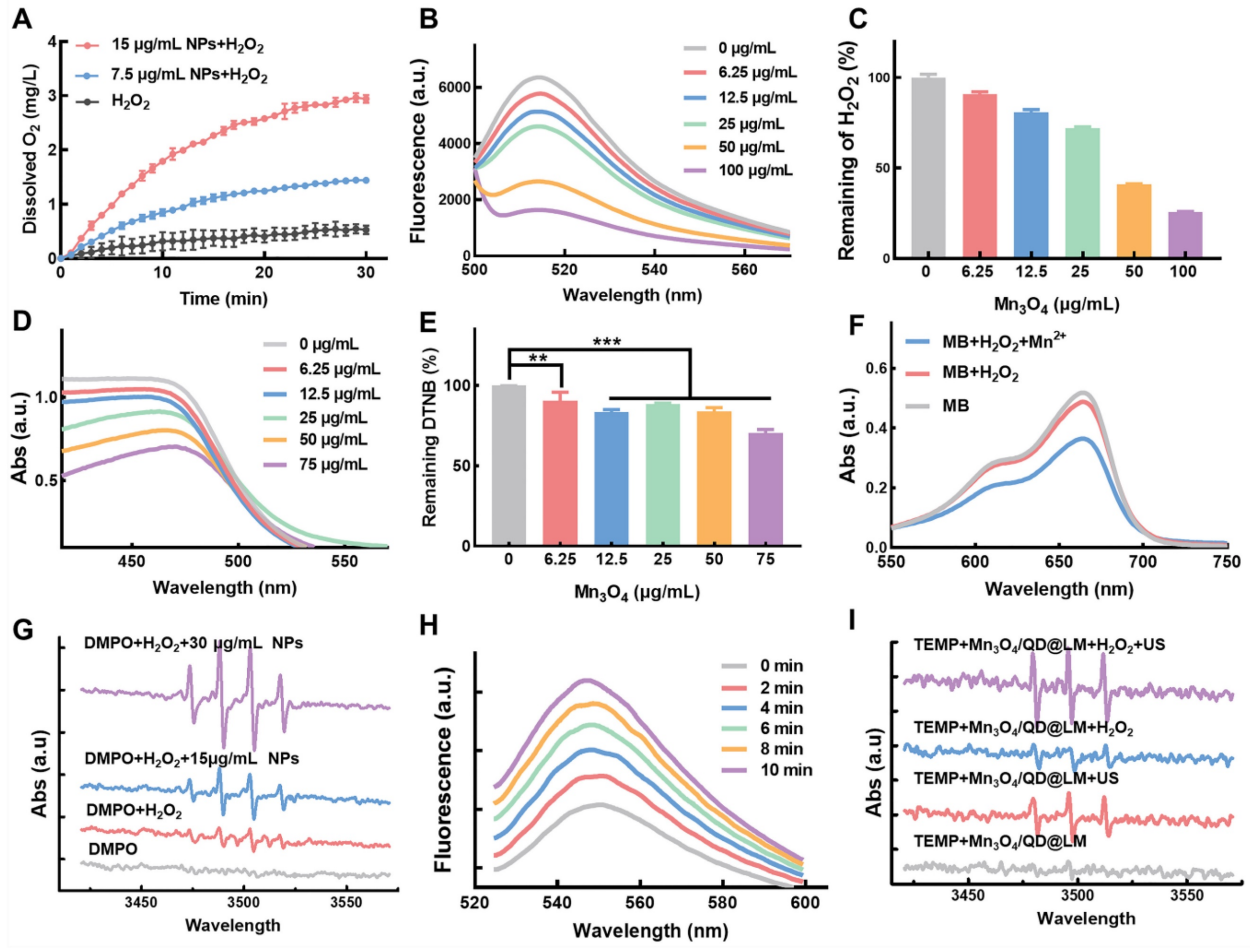
** Mn_3_O_4_/QD@LM exhibits enzyme-like activity and enhances sonodynamic therapy. (A)** Dissolved oxygen levels detected by an oxygen meter after the reaction of Mn_3_O_4_/QD@LM with H_2_O_2_. **(B)** Fluorescence intensity of H_2_O_2_-specific fluorescent probe ROSGreen™ after incubation with Mn_3_O_4_/QD@LM at different concentrations. **(C)** H_2_O_2_ depletion capacity of Mn_3_O_4_/QD@LM at different concentrations, evaluated using the ROSGreen™ probe. **(D)** Absorbance of DTNB after incubation of different concentrations of Mn_3_O_4_/QD@LM with 4 mM GSH. **(E)** Quantification of remaining DTNB after incubation with different concentrations of Mn_3_O_4_/QD@LM with 4 mM GSH. **(F)** Detection of ∙OH generated by Mn_3_O_4_/QD@LM via a Fenton-like reaction using the MB decolorization assay. **(G)** Detection of ∙OH generated by Mn_3_O_4_/QD@LM via a Fenton-like reaction using ESR spectroscopy. **(H)** Detection of ^1^O_2_ generated by Mn_3_O_4_/QD@LM using the SOSG fluorescent probe after different ultrasonic irradiation times. **(I)** Detection of ^1^O_2_ generated by Mn_3_O_4_/QD@LM using ESR spectroscopy under different treatment conditions.

We first assessed the CAT-like activity of Mn_3_O_4_/QD@LM. As CAT can catalyze the breakdown of H_2_O_2_ into H_2_O and O_2_, the CAT-like activity of Mn_3_O_4_/QD@LM can be assessed by measuring O_2_ production and H_2_O_2_ consumption. As shown in **Figure 3A**, the dissolved O_2_ level in the control group (1 mM H_2_O_2_) remained relatively stable over 30 min. In contrast, the O_2_ content in the Mn_3_O_4_/QD@LM + H_2_O_2_ group increased with time and concentration dependently. In addition, the H_2_O_2_-specific fluorescent probe ROSGreen™ was used to detect H_2_O_2_ consumption. The results in **Figure 3B** showed that fluorescence intensity gradually decreased with increasing material concentration. When the material concentration reached 100 μg/mL, the H_2_O_2_ content was only 25% of the initial level (**Figure 3C**). Together, these results demonstrate that the nanoparticles have excellent CAT-like activity. Next, the GPx-like activity of Mn_3_O_4_/QD@LM was assessed using 5,5′-dithiobis (2-nitrobenzoic acid) (DTNB). The reaction product of GSH with DTNB, 2-nitro-5-thiobenzoic acid (TNB), exhibits a characteristic absorption peak at 412 nm. Changes in absorbance at this wavelength reflect GSH levels. As shown in **Figures 3D-E**, the absorption peak at 412 nm steadily decreased with increasing concentrations of Mn_3_O_4_/QD@LM, which indicated progressively greater GSH consumption. These results demonstrate that Mn_3_O_4_/QD@LM can deplete GSH, thereby suppressing the antioxidant capacity of cancer cells, which provides a basis for enhancing ROS-mediated cytotoxicity.

The redox reaction between Mn_3_O_4_/QD@LM and GSH can induce Mn^2+^ generation. The ∙OH produced via the Mn^2+^/H_2_O_2_ Fenton-like reaction can decolorize methylene blue (MB), reducing its absorbance. Therefore, ∙OH generation was first detected using the MB decolorization assay. As shown in **Figure 3F**, the MB solution alone showed the highest absorbance. Adding H_2_O_2_ alone caused no significant change in absorbance. In contrast, the MB absorbance was obviously decreased by the supernatant from nanoparticles pretreated with GSH. This indicates that Mn_3_O_4_/QD@LM releases Mn^2+^ upon reaction with GSH, and the Mn^2+^ subsequently generates ∙OH via Fenton-like reactions. To directly observe ∙OH production, the ∙OH capture agent 5,5-dimethyl-1-pyrroline N-oxide (DMPO) was used, and signal changes were detected by ESR spectroscopy. The ESR spectra in **Figure 3G** show weak signals for the DMPO group and the DMPO + H_2_O_2_ group. Conversely, the Mn_3_O_4_/QD@LM + GSH group exhibited a strong signal.

Next, the sonodynamic effect of Mn_3_O_4_/QD@LM was further assessed. As shown by the fluorescence spectra in **Figure 3H**, the fluorescence intensity of the ¹O_2_-specific fluorescent probe SOSG exhibited a significant time-dependent increase with prolonged ultrasound exposure. Additionally, ESR spectroscopy was used to detect ^1^O_2_ generation by monitoring signal changes of the ^1^O_2_ capture agent 2,2,6,6-tetramethylpiperidine (TEMP) under different conditions. The ESR spectra in **Figure 3I** show that the TEMP + Mn_3_O_4_/QD@LM group produced only a weak background signal. In contrast, ultrasound treatment significantly enhanced the characteristic triplet splitting signal. The strongest signal was observed in the TEMP + Mn_3_O_4_/QD@LM + H_2_O_2_ + US group. This result demonstrated that H_2_O_2_ significantly enhances ^1^O_2_ generation by Mn_3_O_4_/QD@LM. This enhancement is attributed to the CAT-like activity of the Mn_3_O_4_ NPs, which continuously supplies O_2_ by catalyzing H_2_O_2_ decomposition, thereby improving SDT efficiency.

In addition, the stability of the Mn_3_O_4_/QD@LM nanoplatform were assessment. We measured the size and polydispersity index (PDI) of Mn_3_O_4_/QD@LM in three different solutions of water, PBS buffer, and RPMI 1640 complete medium containing 10% FBS over a 7-day period. The results showed that the size and PDI of Mn_3_O_4_/QD@LM did not show significant fluctuations over time in the three typical liquid environments mentioned above, indicating that there was no significant aggregation or sedimentation of the Mn_3_O_4_/QD@LM (**Figure S2**). This good stability indicates that Mn_3_O_4_/QD@LM maintains good dispersibility in both saline and complex biological protein-rich media. This result provides an important guarantee for the subsequent therapeutic application of Mn_3_O_4_/QD@LM.

### Therapeutic efficacy of Mn_3_O_4_/QD@LM *in vitro*

Efficient cellular uptake of nanoparticles is essential for their intended function. Dil-labeled nanoparticles (Dil@LM) were co-incubated with colon cancer CT26 cells for different durations (0 - 8 h) and then observed via fluorescence imaging. Confocal microscopy images revealed punctate red fluorescence signals around the cell membrane after 2 h of incubation, indicating the initiation of nanoparticle internalization. By 8 h, the red fluorescence intensity reached its maximum, showing dense fluorescence throughout the cytoplasm. (**Figure S3A**) Quantitative flow cytometry analysis further demonstrated that nanoparticle uptake was highly time-dependent. In the 8 h group, 98.4% of cells exhibited strong fluorescence signals, confirming continuous and accumulative uptake of the nanoparticles (**Figure S3B-C**). The flow cytometry results were consistent with the fluorescence observations, indicating that CT26 cells effectively internalize nanoparticles over time. These findings provide a cellular basis for subsequent nanoparticles to exert enzymatic catalytic effects.

**Figure 4:**
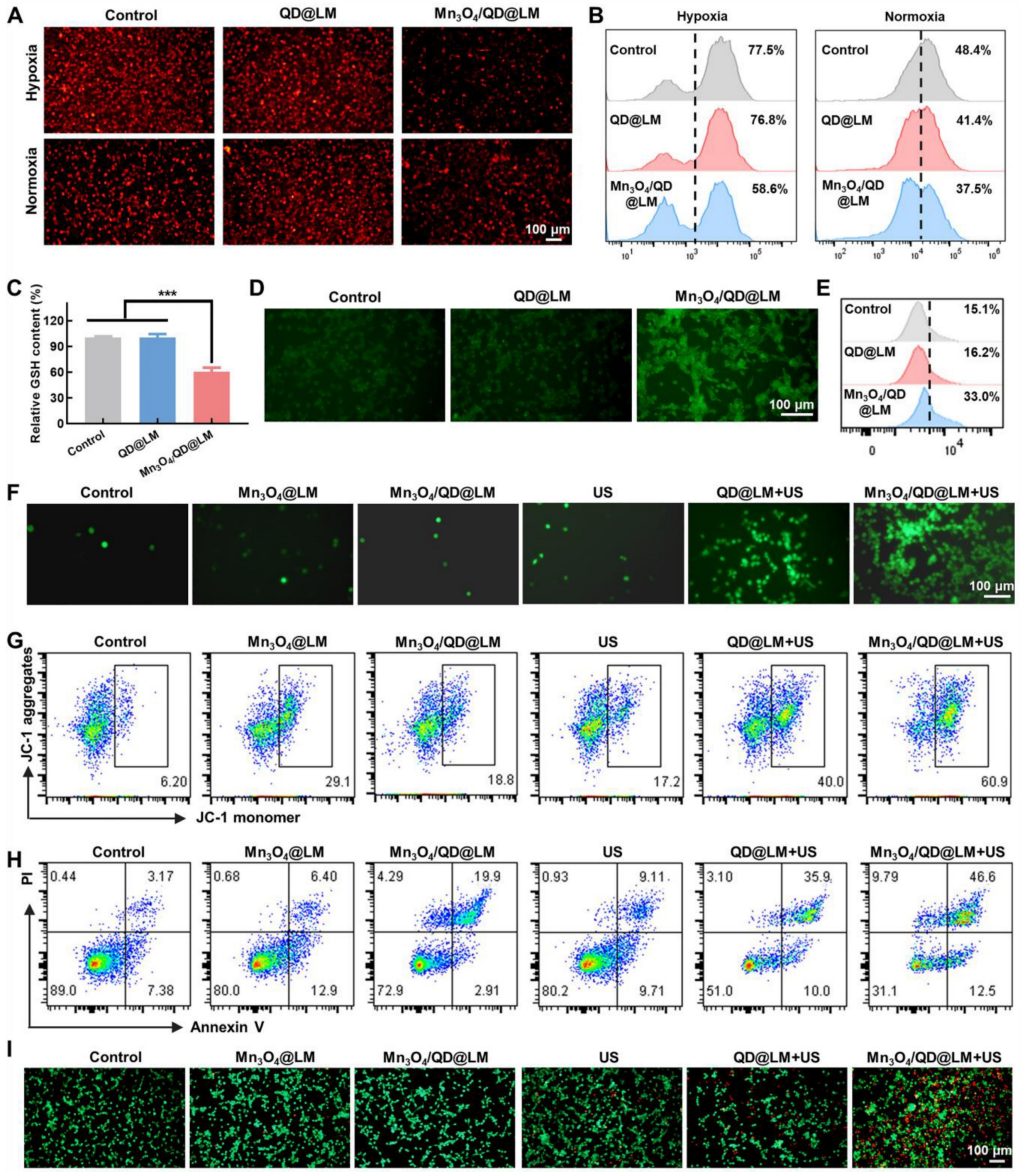
** Therapeutic efficacy of Mn_3_O_4_/QD@LM *in vitro*. (A)** Fluorescence images of cells stained with the O_2_-quenching probe [Ru(dpp)_3_]Cl_2_ under normoxia and hypoxia conditions for different treatment groups. **(B)** Intracellular O_2_ levels were assessed by flow cytometry post-treatment (n = 3). **(C)** Intracellular GSH content measured after various treatments (n = 3). **(D)** Fluorescence images of CT26 cells stained with the HPF following different treatments. **(E)** Evaluation of ∙OH levels by flow cytometry after different treatments (n = 3). **(F)** Fluorescence images of CT26 cells stained with the DCFH-DA following different treatments. **(G)** Representative scatter plots of MMP in CT26 cells evaluated by flow cytometry under different treatments. **(H)** Quantification of apoptosis in CT26 cells using flow cytometry after various treatments. **(I)** Fluorescence images of calcein-AM/PI-stained CT26 cells after different treatments. Statistical comparisons were performed by one-way ANOVA. Statistically significant difference: *p < 0.05, **p < 0.01, and ***p < 0.001.

To investigate the CAT-like activity of Mn_3_O_4_/QD@LM at the cellular level, a hypoxic environment was simulated using the hypoxia-inducing agent desferrioxamine combined with liquid paraffin sealing. Intracellular O_2_ concentration in CT26 cells was detected using the O_2_-quenched red fluorescent probe [Ru(dpp)_3_]Cl_2_. As shown in **Figure 4A**, compared to the control group, the QD@LM group showed no significant change in fluorescence intensity under either oxygen condition. In contrast, the Mn_3_O_4_/QD@LM group exhibited a significant decrease in red fluorescence intensity under both normoxia and hypoxia. Flow cytometry quantification results (**Figure 4B**) further confirm these findings that the CAT-like activity of Mn_3_O_4_ NPs effectively increases intracellular oxygen content. Subsequently, the effect of the nanomaterials on cellular GSH levels (GPx-like activity) was assessed using a GSH quantification kit. As presented in **Figure 4C**, GSH content in the Mn_3_O_4_/QD@LM group significantly decreased to 61.8% of the control level. This result indicates that the GPx-like activity of Mn_3_O_4_ NPs effectively depletes intracellular GSH, thus laying the foundation for enhancing the effect of subsequent SDT. The redox reaction between Mn_3_O_4_/QD@LM and GSH can trigger the release of Mn^2+^, which undergoes a Fenton-like reaction with H_2_O_2_, resulting in high cytotoxicity· OH. To evaluate the ·OH-producing capability of the Mn_3_O_4_/QD@LM, intracellular ·OH levels were assessed using the specific fluorescent probe hydroxyphenyl fluorescein (HPF). Immunofluorescence staining revealed a substantially enhanced green fluorescence signal in cells treated with Mn_3_O_4_/QD@LM compared to the control and QD@LM groups, indicating elevated ·OH production (**Figure 4D**). The flow cytometry analysis results showed a consistent trend (**Figure 4E**). In summary, Mn_3_O_4_/QD@LM exhibits multifaceted catalytic activities at the cellular level, including CAT-like, GPx-like enzymatic functions, and ·OH generation capacity, thus laying the foundation for its subsequent therapeutic efficacy.

It is currently believed that the main mechanism by which SDT exerts its cytotoxic effects is through the production of large amounts of ROS. ROS is one of the key mediators of cellular signaling and oxidative stress. Excessive ROS can induce tumor cell death. Subsequently, ROS production by Mn_3_O_4_/QD@LM was assessed using the ROS-specific fluorescent probe DCFH-DA. The results showed that the control group exhibited weak fluorescence, indicating low levels of endogenous ROS production in tumor cells. The US group showed no significant change in fluorescence intensity (5.4%), confirming that the mechanical effects of US alone were insufficient to induce significant oxidative stress. The Mn_3_O_4_@LM and Mn_3_O_4_/QD@LM groups also displayed partial green fluorescence (3.51% and 6.67%, respectively). This was attributed to partial ROS generation via a Fenton-like reaction from Mn^2+^ ions released during the dissociation of Mn_3_O_4_. The QD@LM + US group showed a significant increase in fluorescence intensity (16.6%). In contrast, the Mn_3_O_4_/QD@LM + US group demonstrated a synergistically enhanced effect, with the proportion of fluorescence-positive cells reaching 26.1% (**Figure 4F and S4**).

Mitochondria serve as key targets for ROS action, and the collapse of mitochondrial membrane potential (MMP) is a hallmark of apoptosis initiation 26. MMP was detected using JC-1 (**Figure 4G and S5A**): The control group maintained normal MMP (green fluorescence: 9.0 ± 2.5%). Both the Mn_3_O_4_@LM group (24.9 ± 8.6%) and the Mn_3_O_4_/QD@LM group (19.8 ± 4.2%) showed mild membrane damage, suggesting oxidation of mitochondrial lipids by Mn^2+^-mediated ROS. Following ultrasound irradiation, the QD@LM + US group (45.7 ± 6.3%) and especially the Mn_3_O_4_/QD@LM + US group (63.3 ± 6.9%) exhibited significantly enhanced green fluorescence. This indicates sonodynamic effect-induced mitochondrial structural damage, with Mn_3_O_4_ NPs further amplifying this effect.

MMP collapse signifies the initiation of the mitochondrial apoptosis pathway 27. Apoptosis in CT26 cells was quantified using Annexin V-FITC/PI dual staining (**Figure 4H and S5B**): The Mn_3_O_4_/QD@LM + US group achieved a significantly higher apoptosis rate (59.9 ± 1.8%) compared to the US group (19.4 ± 3.3%) and the QD@LM + US group (44.2 ± 1.4%). This difference stems from a synergistic mechanism: QD@LM relies solely on the sonosensitizer to generate ^1^O_2_, resulting in limited apoptosis efficiency. In contrast, the Mn_3_O_4_/QD@LM + US group utilizes the oxygen-generating function of Mn_3_O_4_ NPs to enhance the ^1^O_2_ production level of Ag_2_S QDs, coupled with Mn^2+^-triggered Fenton-like reactions. This creates a positive feedback loop for ROS generation, substantially increasing the apoptosis rate. The cytotoxic effect was further visualized by Calcein-AM/PI staining (**Figure 4I**), the QD@LM + US group showed increased red fluorescence (indicating cell death). The Mn_3_O_4_/QD@LM + US group displayed the strongest red fluorescence, confirming that cascade catalytic reactions induce irreversible cell death.

### Immunogenic cell death triggered by Mn_3_O_4_/QD@LM *in vitro*

**Figure 5:**
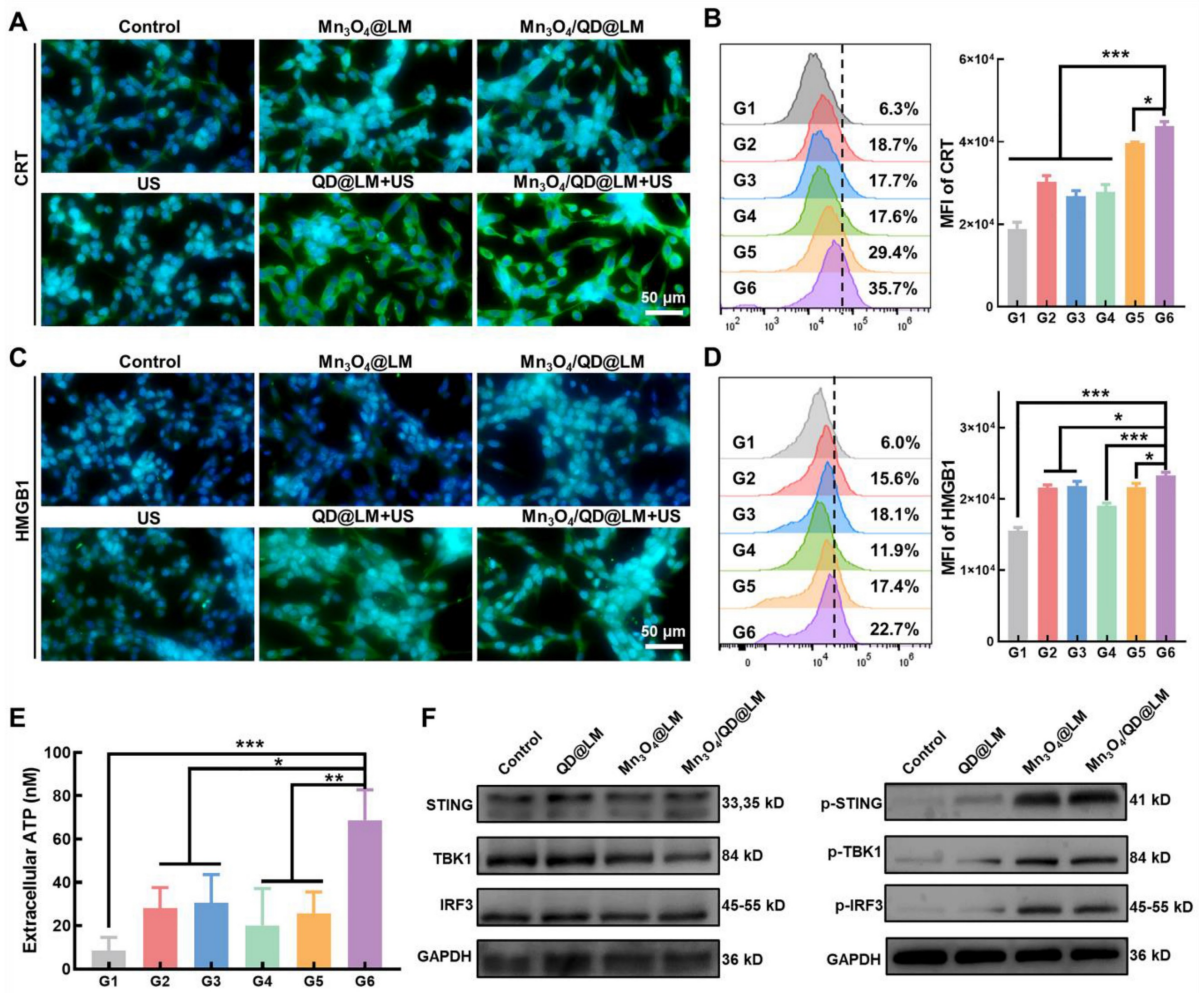
** Immunogenic cell death and STING pathway activation mediated by Mn_3_O_4_/QD@LM *in vitro*. (A)** Immunofluorescence staining of CRT in CT26 cells after different treatments. **(B)** Flow cytometry analysis of the corresponding CRT mean fluorescence intensity (n = 3). **(C)** Immunofluorescence staining of HMGB1 in CT26 cells after various treatments. **(D)** Flow cytometry analysis of the corresponding HMGB1 mean fluorescence intensity (n = 3). **(E)** Extracellular ATP levels of CT26 cells after different treatments (n = 3). **(F)** Western blot analysis of protein levels in the STING pathway (n = 3). G1: Control, G2: Mn_3_O_4_@LM, G3: Mn_3_O_4_/QD@LM, G4: US, G5: QD@LM + US, and G6: Mn_3_O_4_/QD@LM + US. Data are presented as mean ± SD. Statistical comparisons were performed by one-way ANOVA. Statistically significant difference: *p < 0.05, **p < 0.01, and ***p < 0.001.

Previous studies have demonstrated that ROS-mediated SDT can induce ICD in tumor cells. This process is characterized by releasing DAMPs signals, such as the exposure of calreticulin (CRT), adenosine triphosphate (ATP), and high mobility group box 1 (HMGB1), thereby activating the immune response. Therefore, we first evaluated the impact of SDT on CRT and HMGB1 expression. Immunofluorescence imaging (**Figure 5A**) revealed that cells treated with US alone exhibited weak green fluorescence, whereas the Mn_3_O_4_@LM and Mn_3_O_4_/QD@LM groups showed elevated fluorescence, suggesting that Mn^2+^ alone can induce a limited degree of ICD. Notably, the Mn_3_O_4_/QD@LM + US group displayed the strongest green fluorescence, confirming a marked increase in CRT exposure on the surface of CT26 cells. Consistent with these observations, flow cytometric analysis (**Figure 5B**) demonstrated that CRT fluorescence intensity was significantly higher in the Mn_3_O_4_/QD@LM + US group compared to all other groups. Furthermore, immunofluorescence images and flow cytometry analysis of HMGB1 (**Figure 5C-D**) revealed a similar trend. In addition, the Mn_3_O_4_/QD@LM + US group exhibited the highest level of ATP secretion among all treatment groups (**Figure 5E**). Collectively, these results demonstrate that Mn_3_O_4_/QD@LM + US can trigger ICD effectively.

### Mn_3_O_4_/QD@LM can active STING signaling pathway

Mn^2+^ can activate the STING signaling pathway by enhancing the binding affinity of cyclic GMP-AMP synthase (cGAS) to its substrate cGAMP 19. To assess the capacity of Mn_3_O_4_/QD@LM to activate the STING pathway, we performed western blot analysis to detect TBK1, STING, and IRF3 proteins and their phosphorylation levels in CT26 cells after incubation with different nanoparticles. In contrast to the control and QD@LM groups, treatment with Mn_3_O_4_/QD@LM nanoparticles resulted in markedly elevated expression levels of phosphorylated proteins p-TBK1, p-STING, and p-IRF3 (**Figure 5F**). These findings suggest that Mn^2+^ released by Mn_3_O_4_/QD@LM in the TME effectively activates the STING pathway, thereby enhancing the anti-tumor immune response.

### Biocompatibility of Mn_3_O_4_/QD@LM *in vivo*

**Figure 6:**
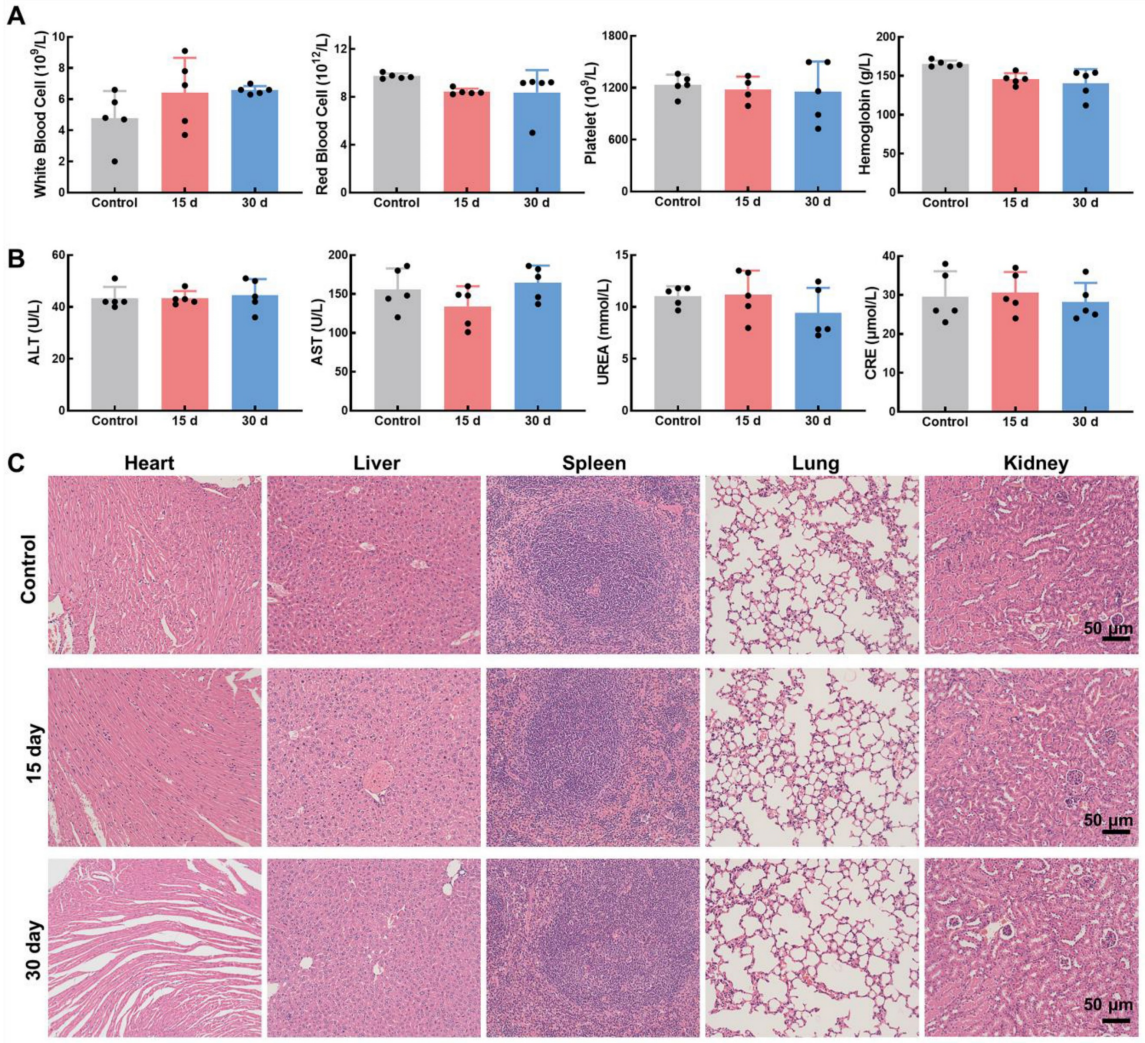
** Biocompatibility of Mn_3_O_4_/QD@LM *in vivo.* (A)** Hematological parameters in mice at various time points following the administration of Mn_3_O_4_/QD@LM or saline (n = 5). **(B)** Blood biochemical parameters in mice at different time points after injection of Mn_3_O_4_/QD@LM or saline (n = 5). **(C)** H&E-stained images of major organs from mice at various time points following the administration of Mn_3_O_4_/QD@LM or saline.

Good biosafety of nanomaterials is a prerequisite for *in vivo* therapeutic applications. To evaluate the *in vivo* biosafety of Mn_3_O_4_/QD@LM, we dynamically monitored changes in liver/kidney function and hematological parameters in mice at multiple time points after intravenous injection of Mn_3_O_4_/QD@LM. Hematological analysis revealed that on day 15 and day 30 post-injection, key blood cell parameters - including white blood cells, red blood cells, platelets, and hemoglobin - in the nanomaterial-injected group remained comparable to those in the control group (**Figure 6A**). This demonstrates that Mn_3_O_4_/QD@LM did not disrupt hematopoietic system homeostasis. The liver and kidneys, being the primary organs for nanoparticle metabolism, are critical for assessing material toxicity. As shown in **Figure 6B**, serum levels of alanine aminotransferase (ALT), aspartate aminotransferase (AST), creatinine, and blood urea nitrogen (BUN) in the experimental group showed no statistically significant differences from the control group on day 15 and day 30. These results indicate that Mn_3_O_4_/QD@LM caused neither hepatocellular membrane damage nor glomerular filtration dysfunction. Furthermore, histopathological evidence in **Figure 6C** showed no pathological alterations in H&E-stained sections of major organs (Heart, Liver, Spleen, Lung, Kidney) after 15 or 30 days of exposure. Collectively, these results demonstrate the minimal biosafety concerns of Mn_3_O_4_/QD@LM, providing a solid foundation for its potential use in *in vivo* therapeutics.

### Tumor targeting and biodistribution of Mn_3_O_4_/QD@LM

**Figure 7:**
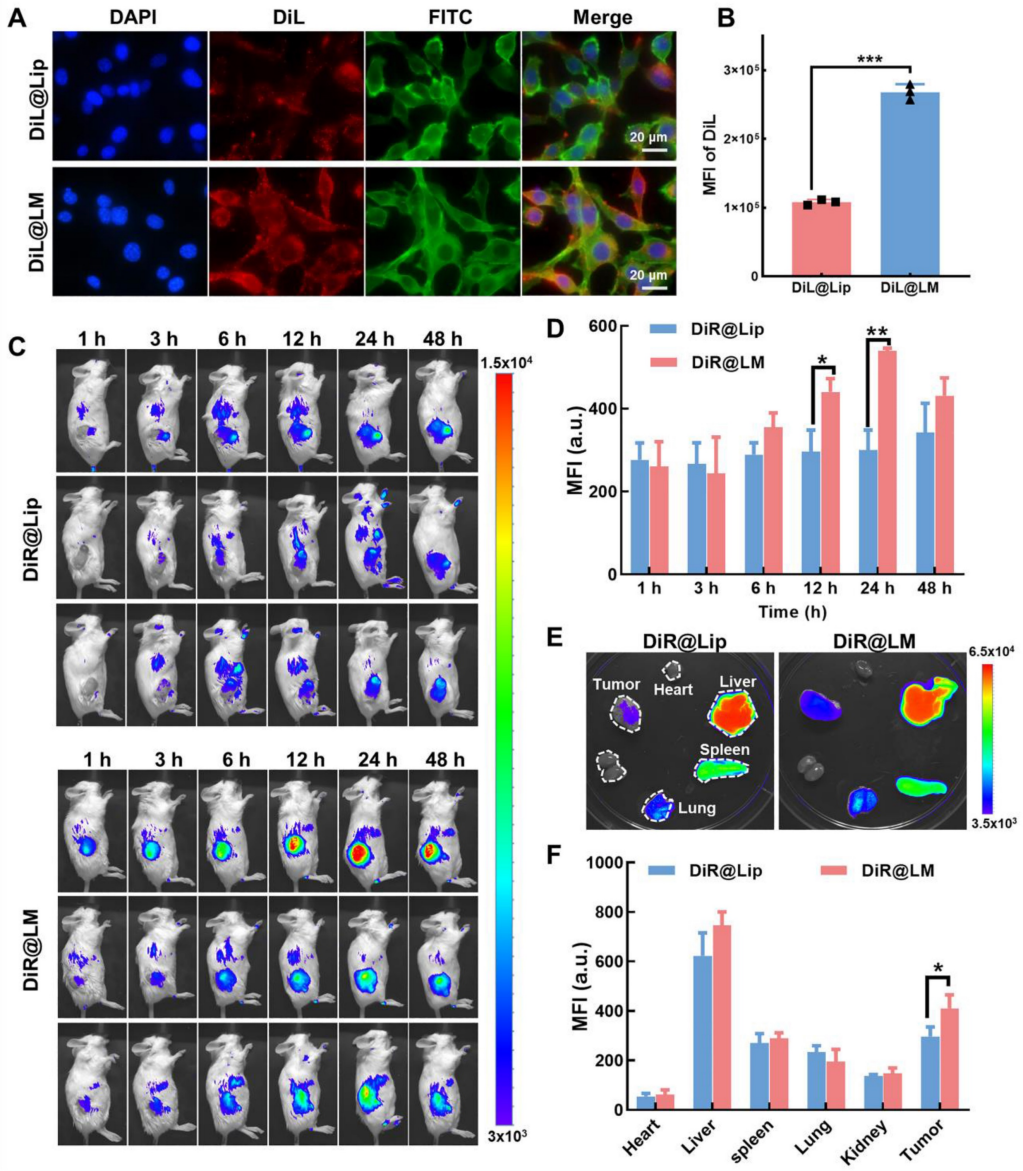
** Tumor targeting and biodistribution of Mn_3_O_4_/QD@LM. (A)** Fluorescence image and **(B)** flow cytometry analysis of CT26 cells following an 8-h incubation with DiL@Lip or DiL@LM (n = 3). **(C)** Dynamic distribution of fluorescence signals in CT26 tumor-bearing mice at the indicated times post-injection of DiR@Lip or DiR@LM, and **(D)** corresponding fluorescence quantification (n = 3). **(E)** Fluorescence images of dissected organs and tumors from CT26 tumor-bearing mice 48 h after intravenous injection of DiR@Lip or DiR@LM, and **(F)** corresponding fluorescence quantification (n = 3). Data are presented as mean ± SD. Statistical comparisons were performed by one-way ANOVA. Statistically significant difference: *p < 0.05, **p < 0.01, and ***p < 0.001.

Effective uptake by target cells is a prerequisite for nanoparticles to work. In recent years, isotype tumor cell membrane coating has become a key strategy to improve the targeting efficiency of nanoparticles. This mechanism relies on natural adhesion molecules and specific surface antigens conserved on the tumor cell membrane to achieve specific binding to homologous tumor cells through "isotype recognition" 28. Several studies have demonstrated that this strategy can significantly enhance the accumulation of nanoparticles at tumor sites 29, 30. First, we prepared DiL-labeled liposomes (DiL@Lip) and biomimetic nanoliposomes incorporating tumor cell membrane (DiL@LM). After incubating CT26 cells with both nanoparticles for 8 h, cell uptake was assessed. As shown in **Figure 7A**, CT26 cells internalized some DiL@Lip after 8 h co-incubation. However, a significantly stronger fluorescence signal was observed in cells incubated with DiL@LM. Flow cytometry analysis (**Figure 7B**) quantitatively confirmed this: the mean fluorescence intensity of the DiL@LM group was 2.65 times higher than that of the DiL@Lip group, further proving that the tumor cell membrane coating enhanced the targeting of nanoparticles.

To investigate the biodistribution and tumor-targeting capabilities of nanoparticles *in vivo*, DiR-labeled DiR@Lip or DiR@LM was administered intravenously to CT26 tumor-bearing mice. As shown in **Figure 7C**, the fluorescence intensity of the tumor site in both groups increased over time, peaking at 24 h post-injection and then gradually decreasing. Importantly, DiR@LM showed significantly enhanced tumor accumulation compared to DiR@Lip. Tumor fluorescence intensity in the DiR@LM group was 1.48-fold higher than in the DiR@Lip group at 12 h, and this targeting advantage further increased to 1.79-fold at 24 h (**Figure 7D**). Subsequently, mice were sacrificed at 48 h post-injection. Major organs and tumor tissue were collected, and the distribution of nanoparticles was analyzed. *Ex vivo* imaging showed the highest fluorescence signal intensity in the liver, consistent with its role as the primary metabolic organ responsible for nanoparticle clearance (**Figure 7E**). Importantly, *ex vivo* tumor imaging further confirmed that the fluorescence intensity of the DiR@LM group was significantly higher than that of the DiR@Lip group (**Figure 7F**). Together, these findings demonstrate that the liposomes incorporated with tumor cell membranes (DiR@LM) have stronger tumor-targeting efficiency compared to uncoated liposomes (DiR@Lip).

### Anti-tumor effect of Mn_3_O_4_/QD@LM *in vivo*

**Figure 8:**
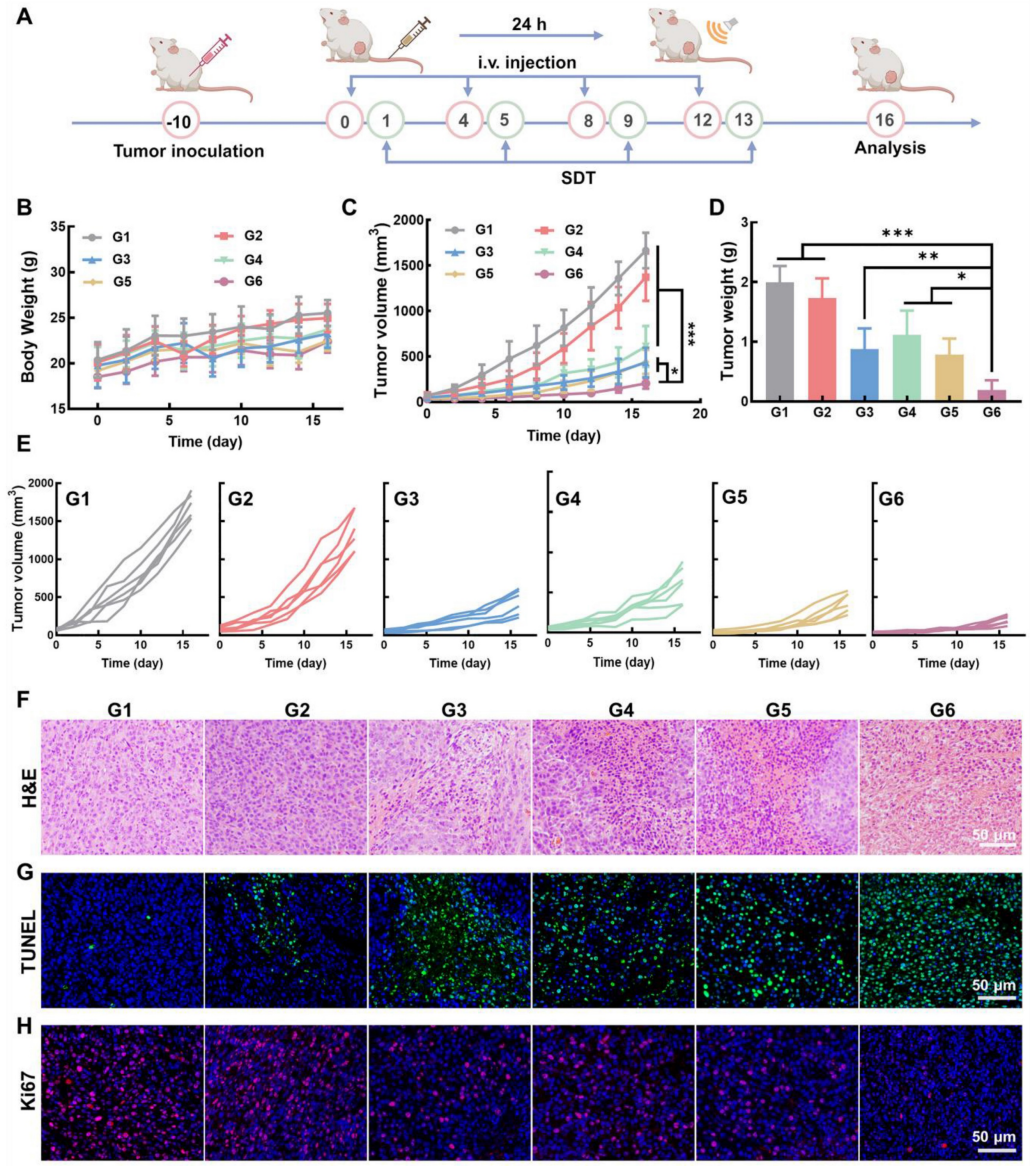
** Anti-tumor effect of Mn_3_O_4_/QD@LM *in vivo.* (A)** Schematic diagram of the antitumor experiment *in vivo*. **(B)** Body weight of mice in each treatment group (n = 5). **(C)** Average tumor volume of mice following different treatments (n = 5). **(D)** Tumor weight measured at the end of the experiment for each group (n = 5). **(E)** Tumor growth curves of individual animals across treatment groups. **(F)** H&E staining, **(G)** TUNEL immunofluorescence staining, and **(H)** Ki67 immunofluorescence staining images of tumor tissue of mice in different treatment groups. G1: Control, G2: US, G3: Mn_3_O_4_@LM, G4: QD@LM + US, G5: Mn_3_O_4_/QD@LM, and G6: Mn_3_O_4_/QD@LM + US. Data are presented as mean ± SD. Statistical comparisons were performed by one-way ANOVA. Statistically significant difference: *p < 0.05, **p < 0.01, and ***p < 0.001.

In order to evaluate the *in vivo* therapeutic effect of Mn_3_O_4_/QD@LM, CT26 tumor-bearing mice were randomly divided into six groups: G1: Control; G2: US; G3: Mn_3_O_4_@LM; G4: QD@LM + US; G5: Mn_3_O_4_/QD@LM; G6: Mn_3_O_4_/QD@LM + US. The treatment process is shown in **Figure 8A**. Mice are injected with different nanomaterials through the tail vein. At 24 h post-injection, the ultrasound treatment group received US irradiation (1.5 W/cm^2^, 3 min). Mouse body weight and tumor volume were recorded every two days throughout the experiment.

As shown in **Figure 8B**, the average body weights across all groups showed no significant difference, which demonstrates that the nano-system did not induce metabolic disorders or systemic toxicity during treatment. Tumor volume and tumor weight measurements (**Figure 8C-E**) revealed that the US group showed only a weak inhibitory effect on tumor growth. In contrast, both the Mn_3_O_4_@LM and Mn_3_O_4_/QD@LM groups showed stronger antitumor effects. This is primarily attributed to the Fenton-like activity of Mn_3_O_4_ NPs within the tumor microenvironment. The tumor volume in the QD@LM + US group was 46.8% of that in the control group, indicating the limitations of a single treatment modality. Tumor growth inhibition was most significant in the Mn_3_O_4_/QD@LM + US group, with a tumor suppression rate of 87.6% after 16 days of treatment. Furthermore, the *ex vivo* tumor image in **Figure S6** visually confirmed the pronounced tumor suppression effect in the Mn_3_O_4_/QD@LM + US group.

To further evaluate the tumor inhibitory effect of Mn_3_O_4_/QD@LM, histopathological analysis was performed on tumor tissue. H&E staining results showed that the tumor cells in the control group maintained normal morphology, and the nucleus was intact. The Mn_3_O_4_/QD@LM + US group displayed characteristic apoptotic morphological changes and the largest areas of necrosis (**Figure 8F**). Subsequently, TUNEL staining was used to analyze cell apoptosis (**Figure 8G**). Compared to the control group, an enhanced green fluorescence signal was observed in both the Mn_3_O_4_@LM and Mn_3_O_4_/QD@LM groups, which confirmed Mn_3_O_4_ NPs can induce tumor cell death through their Fenton-like effect. The strongest green fluorescence was detected in the Mn_3_O_4_/QD@LM+US group, indicating that the apoptosis in this group was the most severe, indicating the superior efficacy of synergistic treatment. Furthermore, cell proliferation activity was assessed by detecting the expression level of the nuclear proliferation marker Ki67 (**Figure 8H**). Immunofluorescence results showed that the control group had the strongest Ki67-positive red fluorescence signal. The lowest signal intensity was observed in the Mn_3_O_4_/QD@LM + US group.

### Mechanistic insights into the anti-tumor immune response of Mn_3_O_4_/QD@LM

**Figure 9:**
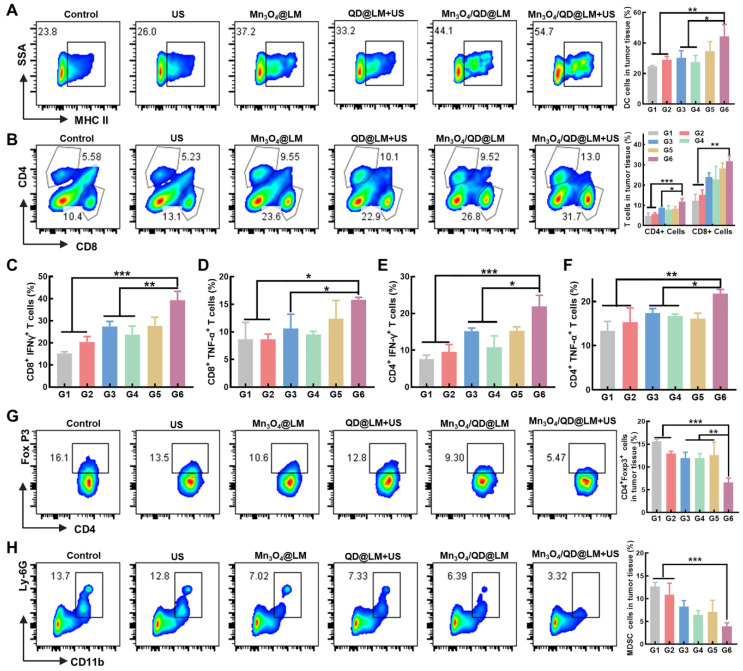
** Mechanism of anti-tumor immune response of Mn_3_O_4_/QD@LM. (A)** Representative flow cytometry plots and quantitative analysis of mature DCs within the tumor (n = 3). **(B)** Representative flow cytometry plots and quantitative analysis of T cells within the tumor (n = 3). **(C)** Quantitative analysis of CD8⁺IFN-γ⁺ T cells in tumor tissues (n = 3). **(D)** Quantitative analysis of CD8⁺TNF-α⁺ T cells in tumor tissues (n = 3). **(E)** Quantitative analysis of CD4⁺IFN-γ⁺ T cells in tumor tissues (n = 3). **(F)** Quantitative analysis of CD4⁺TNF-α⁺ T cells in the tumor (n = 3). **(G)** Representative flow cytometry plots and quantitative analysis of Tregs in tumor tissues (n = 3). **(H)** Representative flow cytometry plots and quantitative analysis of MDSCs in tumor tissues (n = 3). G1: Control, G2: US, G3: Mn_3_O_4_@LM, G4: QD@LM+US, G5: Mn_3_O_4_/QD@LM, and G6: Mn_3_O_4_/QD@LM +US. Data are presented as mean ± SD. Statistical comparisons were performed by one-way ANOVA. Statistically significant difference: *p < 0.05, **p < 0.01, and ***p < 0.001.

To gain deeper insight into the immune response elicited by Mn_3_O_4_/QD@LM during treatment, we analyzed immune cell subsets within tumor tissues harvested from mice post-treatment. DCs are central regulators of adaptive immunity. Their maturation state directly determines the strength of the anti-tumor immune response. Mature DCs highly express major histocompatibility complex class II molecules (MHC II) and co-stimulatory molecules. They present tumor antigens to T cells, activating specific cytotoxic T lymphocyte (CTLs) responses. First, we assessed the proportion of mature DCs (CD45⁺CD11c⁺MHC II⁺) in tumor tissues using flow cytometry. As shown in **Figure 9A**, the combined Mn_3_O_4_/QD@LM + US treatment group exhibited significantly higher MHC II expression (44.2 ± 7.9%) on DCs compared to the Mn_3_O_4_@LM group (34.0 ± 6.3%) and the QD@LM + US group (27.3 ± 4.4%). This highlights the distinct advantage of the combined therapy in promoting DC maturation. Acting as immune system "sentinels", mature DCs drive the activation and expansion of tumor-infiltrating lymphocytes. Within the anti-tumor immune response, CD8⁺ T cells serve as key effector cells, capable of specifically recognizing and mediating tumor cell lysis. CD4⁺ T cells primarily provide helper functions. By secreting cytokines like IL-2 and IFN-γ, they enhance CD8⁺ T cell cytotoxicity and promote sustained DC maturation, forming a positive feedback immune regulatory loop 31. Flow cytometric quantification revealed that the proportions of both CD8⁺ T cells and CD4⁺ T cells within the tumor tissue were significantly higher than in other groups (**Figure 9B**). Specifically, the cytotoxic CD8⁺ T cell subset reached 31.8 ± 2.8%, representing a 2.6-fold increase over the Control group. The helper CD4⁺ T cell subset also significantly increased to 11.7 ± 1.6%, a 2.5-fold increase over the Control group. These results collectively demonstrate that Mn_3_O_4_/QD@LM + US treatment triggers a robust anti-tumor immune effect. As core mediators of adaptive immunity, the cytokines TNF-α and IFN-γ play crucial roles in anti-tumor responses. Flow cytometric analysis of cytokine-secreting cell proportions revealed that the Mn_3_O_4_/QD@LM + US group exhibited the highest percentages of CD8⁺IFN-γ⁺ T cells (39.3 ± 4.0%) and CD8⁺TNF-α⁺ T cells (15.8 ± 0.4%) within the tumor compared to all other groups **(Figures 9C-D and S7A-B)**. Similarly, the proportions of tumor-infiltrating CD4⁺IFN-γ⁺ T cells (21.9 ± 3.0%) and CD4⁺TNF-α⁺ T cells (21.8 ± 1.0%), which are vital for regulating adaptive immunity, were also significantly higher in the Mn_3_O_4_/QD@LM + US group than in other groups **(Figures 9E-F and S7C-D)**.

Within the complex regulatory network of tumor immunotherapy, the dynamic balance between CTLs and immunosuppressive cells directly dictates the strength and durability of the anti-tumor immune response. Tregs can accumulate in TME, impairing effector T cell function 32. Furthermore, MDSCs present in the TME are key mediators of tumor immune tolerance, partly by restricting CD8⁺ T cell infiltration into tumor sites 33. To determine if the combined strategy could maximize the anti-tumor immune response, we next measured the proportions of these two immunosuppressive cell types in mouse tumors. As shown in **Figures 9G-H**, statistical analysis indicated that Mn_3_O_4_/QD@LM + US treatment significantly reduced the proportion of Tregs (to 6.5 ± 0.9% vs. 15.6 ± 0.4% in the control group) and MDSCs (to 3.9 ± 0.8% vs. 12.7 ± 0.9% in the control group).

These *in vivo* results collectively demonstrate that Mn_3_O_4_/QD@LM + US therapy can drive immune activation and amplify tumor antigen-specific killing by enhancing IFN-γ and TNF-α secretion from CD8⁺ and CD4⁺ T cells. At the same time, it can alleviate immunosuppressive constraints by substantially reducing Tregs and MDSCs infiltration, thereby weakening their inhibition of effector T cells and disrupting the vicious cycle of tumor immune escape. These findings indicate that Mn_3_O_4_/QD@LM + US provides a novel synergistic strategy for overcoming resistance to immunotherapy in solid tumors.

## Conclusion

To address the limitations of SDT in the hypoxic tumor microenvironment and high levels of GSH, this study designed and constructed a bio-mimetic liposome platform which loads ultra-small Mn_3_O_4_ NPs and Ag_2_S QDs to enhance the efficacy of sonodynamic-immunotherapy for colorectal cancer. The nanosystem demonstrated excellent CAT-like and GPx-like activities *in vitro*, enabling self-supply of oxygen and depletion of GSH, thereby alleviating tumor hypoxia and reducing the reductive microenvironment, which significantly improved the SDT efficiency. Moreover, it mediated a Fenton-like reaction to generate ·OH, further synergizing tumor cell killing. Cellular experiments revealed that upon US activation, Mn_3_O_4_/QD@LM exhibited remarkable antitumor effects *in vitro*. In a CT26 tumor-bearing mouse model, the platform achieved high tumor-specific accumulation through homologous targeting. Combined with ultrasound treatment, it achieved a tumor inhibition rate of 87.6%, and effectively remodeled the tumor immune microenvironment by promoting CD8⁺ T cell infiltration and downregulating Tregs and MDSCs. These findings provide a new and promising paradigm for improving the efficacy of sonodynamic-immunotherapy.

## Supplementary Material

Supplementary figures.

## Data Availability

The data that support the findings of this study are available from the corresponding author upon reasonable request.

## Abbreviations

SDT: sonodynamic therapy
ROS: reactive oxygen species
Ag_2_S QDs: Ag_2_S quantum dots
Tregs: regulatory T cells
MDSCs: myeloid-derived suppressor cells
GSH: glutathione
TME: tumor microenvironment
ICD: immunogenic cell death
CAT: catalase
GPx: glutathion peroxidase
∙OH: hydroxyl radicals
DTNB: 5,5'-dithiobis (2-nitrobenzoic acid)
ESR: electron spin resonance
MB: methylene blue
^1^O_2_: singlet oxygen
TEM: transmission electron microscopy
XPS: X-ray photoelectron spectroscopy
CM: cell membranes
DLS: dynamic light scattering
DMPO: 5,5-dimethyl-1-pyrroline N-oxide
TEMP: 2,2,6,6-tetramethylpiperidine
MMP: mitochondria membrane potential
DAMPs: damage-associated molecular patterns
CRT: calreticulin
HMGB1: high-mobility group box 1
ALT: alanine aminotransferase
AST: aspartate aminotransferase
BUN: blood urea nitrogen
MHC II: major histocompatibility complex class II molecules
CTLs: cytotoxic T lymphocyte
