# Supplementary material for: Bioinspired Nanoplatform Potentiates Sonodynamic Immunotherapy by Remodeling the Antioxidant Tumor Microenvironment and Activating STING pathway
Source: Theranostics. 2026 Apr 16;16(11):6145–63. doi: 10.7150/thno.129765 (PMC13142239; doi:10.7150/thno.129765)
Supplement: Supplementary file 1 — Supplementary figures. [file thnov16p6145s1.pdf]

Supplementary Information

Bioinspired nanoplatform potentiates sonodynamic immunotherapy by remodeling the antioxidant tumor microenvironment and activating STING pathway

Yuanyuan Zhang[1#], Wenxiang Zhu[2#], Kaimin Li[3#], Yun Xie[1], Zhichao Deng[4], Yuanyuan Zhu[4], Bowen Gao[4], Chenxi Xu[4], Junlong Fu[4], Mingzhen Zhang[4*], Xiaoliang Zheng[5*], and Haifeng Zhang[6,7*]

Affiliations:

1. The Clinical Laboratory, Northwest Women's and Children's Hospital, Xi'an, Shaanxi, 710061, China
2. Nanozyme Laboratory in Zhongyuan, Henan Academy of Innovations in Medical Science, Zhengzhou, Henan, 451163, China
3. School of Biological and Medical Engineering, Beijing University of Aeronautics and Astronautics, Beijing, 100191, China
4. School of Basic Medical Sciences, Xi'an Jiaotong University, Xi'an, Shaanxi, 710061, China
5. School of Laboratory Medicine and Bioengineering, Hangzhou Medical College, Hangzhou, Zhejiang, 310053, China
6. Department of Pathology, School of Basic Medical Sciences, Xi'an Jiaotong University, Xi'an, Shaanxi, 710061, China
7. Institute of Genetics and Developmental Biology, Translational Medicine Institute, Xi'an Jiaotong University, Xi'an, Shaanxi, 710061, China

[#] The authors contribute equally to this work

Mingzhen Zhang (ORCID: 0000-0002-4686-6526)
Email address: mzhang21@xjtu.edu.cn

Xiaoliang Zheng (ORCID: 0000-0002-7955-7455)
Email address: Zhengxl@hmc.edu.cn

Haifeng Zhang (ORCID: 0000-0003-4749-8953)
Email address: zhf19841011@xjtu.edu.cn

Materials and Methods

Hydrogenated Soybean Phosphotidylcholine (HSPC), 1,2-distearoyl-sn-glycero-3-phosphoethanolamine-N-[methoxy(polyethylene glycol)-2000] (DSPE-PEG2000), Cholesterol were purchased from RuiXi Biochemical Co., Ltd; Diethyldithiocarbamic acid silver salt (Ag(DDTC), 98%) was bought from Aladdin Industrial Co., Ltd.; Manganese(II)2,4-pentanedionate, Octadecene (ODE, 95%), Methylene Blue were purchased from Macklin Biochemical Co., Ltd; ROSGreen^{TM} H_2O_2 probe was purchased from Maokang Co., Ltd; 2,2,6,6-tetramethylpiperidine (TEMP, 95%), 5,5-dimethyl-1-pyrroline N-oxide (DMPO) and 2,7-dichlorofluorescin diacetate (DCFH-DA, 97%) were purchased from Sigma-Aldrich Co., Ltd; The following reagents were procured from Beyotime Biotechnology Co., Ltd: Singlet Oxygen Sensor Green (SOSG), Mitochondrial Membrane Potential Assay Kit (JC-1), Reactive Oxygen Species Assay Kit, and the Membrane and Cytosol Protein Extraction Kit. The FITC Annexin V Apoptosis Detection Kit was sourced from BD Biosciences. All antibodies for flow cytometry were obtained from BioLegend. All chemicals were of analytical grade and used without further purification.

Determination of Encapsulation Efficiency of Mn_3O_4 NPs and Ag_2S QDs in Mn_3O_4/QD@LM

The encapsulation efficiencies (EE) of Mn_3O_4 NPs and Ag_2S QDs were determined by ICP-MS. After synthesizing Mn_3O_4/QD@LM, 500 μL of the product was digested with concentrated nitric acid-perchloric acid. Excess acid was evaporated, the solution was fixed to volume with 2% dilute nitric acid, filtered through a 0.22 μm membrane, and detected by ICP-MS.

Based on the detected Mn^{2+} and Ag^{+} concentrations, the mass of these ions in the 500 μL sample was calculated, and then converted to the total mass in the entire product volume. Using the relative molecular masses of Mn_3O_4 (190.89 g/mol) and Ag_2S (247.80 g/mol), the total Mn^{2+} and Ag^{+} masses

were converted to the actual mass of encapsulated Mn_3O_4 NPs and Ag_2S QDs, respectively. EE was calculated with the formula:

$$EE\ (\%) = \frac{M_{encap}}{M_{feed}}$$

M_{encap} = mass of Mn_3O_4 NPs or Ag_2S QDs encapsulated in the liposomes

M_{feed} = initial feeding mass of Mn_3O_4 NPs or Ag_2S QDs during the synthesis process.

Evaluation of the Catalase-like Activity of Mn_3O_4/QD@LM at the Cellular Level

The intracellular oxygen levels in CT26 cells were quantitatively assessed using the oxygen-sensitive fluorescent probe $[Ru(dpp)_3]Cl_2$ to evaluate the oxygen-modulating capacity of the nanoparticles under both normoxic and simulated hypoxic conditions. CT26 cells were seeded in 6-well plates at a density of 5×10^5 cells per well and cultured until reaching 80% confluence. Two experimental conditions were established. The normoxic group was maintained under standard culture conditions. The hypoxic group was treated with deferoxamine (DFO; 30 μM) and covered with a 3 mm layer of liquid paraffin to establish a combined chemical-physical hypoxia model.

Different types of nanoparticles (Control, QD@LM, Mn_3O_4/QD@LM) were added to their respective wells, and the cells were incubated for another 6 h. After treatment, the culture medium was removed, and the cells were washed three times with pre-cooled PBS. Then, 2 mL of serum-free medium containing 30 μM $[Ru(dpp)_3]Cl_2$ probe was added to each well, followed by incubation in the dark for 30 min. After removing the probe-containing medium, the cells were washed three times with pre-cooled PBS. Fluorescence images were acquired using a fluorescence microscope under consistent exposure settings, with multiple randomly selected fields captured per well.

Evaluation of the Glutathione Peroxidase-like Activity of Mn_3O_4/QD@LM at the Cellular Level

The intracellular GSH level in CT26 cells was measured using a commercial GSH quantification kit. CT26 cells were seeded in 6-well plates at a density of 5×10^5 cells per well and cultured until they reached 80% confluence. The cells were then treated with different types of nanoparticles (Control, QD@LM, Mn_3O_4/QD@LM) for 6 h. After incubation, the culture medium was removed, and the cells were collected and disrupted by ultrasonication. GSH quantification was performed according to the manufacturer's instructions. Briefly, 100 μL of cell lysate was mixed with an equal volume of Reagent 1, and the mixture was centrifuged at 3500 rpm for 10 min. The supernatant was collected for analysis. The GSH standard provided in the kit was diluted to various concentrations. Then, 100 μL of precipitant was added to the blank well, 100 μL of standard solution was added to the standard wells, and 100 μL of the supernatant was added to the sample wells. Subsequently, 25 μL of reagent 2 and 100 μL of reagent 3 were added to each well. After incubating in the dark for 10 min, the absorbance at 405 nm was measured.

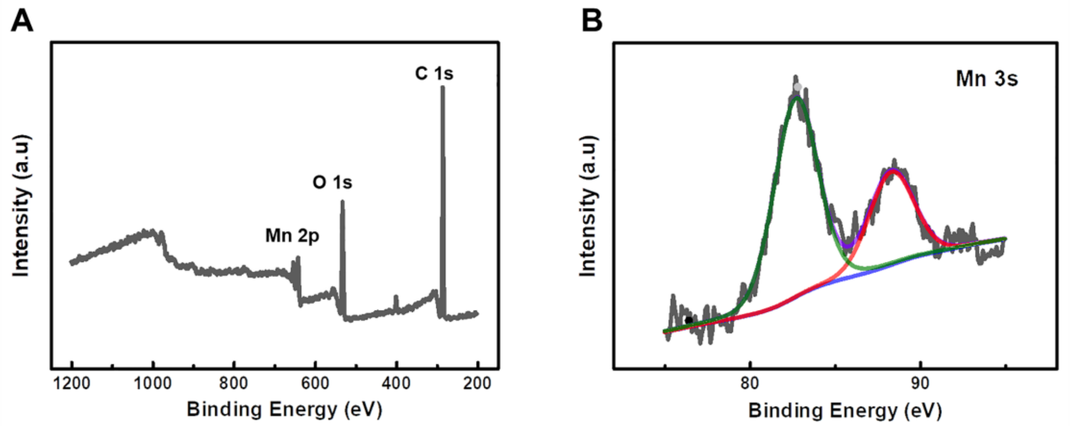

Figure S1. **XPS spectra of Mn_3O_4 NPs.** (A) XPS spectra for the C 1s peak of Mn_3O_4 NPs. (B) XPS spectra for the Mn 3s peak of Mn_3O_4 NPs.

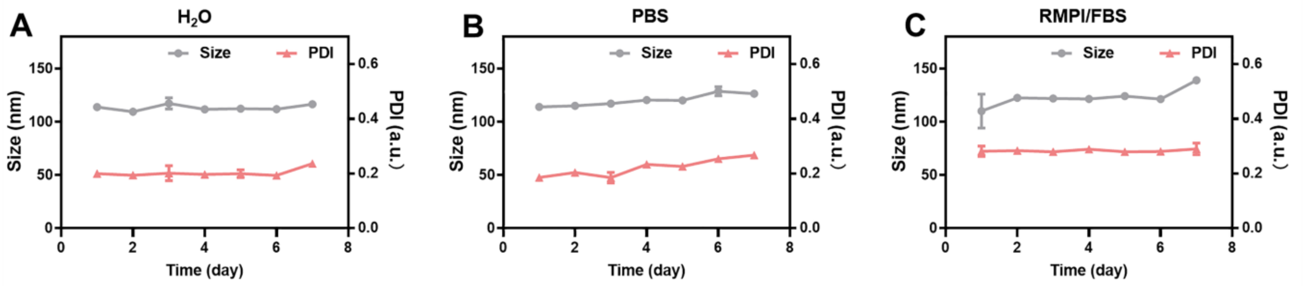

Figure S2 Stability of Mn_3O_4/QD@LM. Changes in hydrated particle size and polydispersity index (PDI) over time of Mn_3O_4/QD@LM in water (A), PBS (B), and RPMI 1640 medium with 10% FBS. (n = 3). Data are presented as mean ± SD

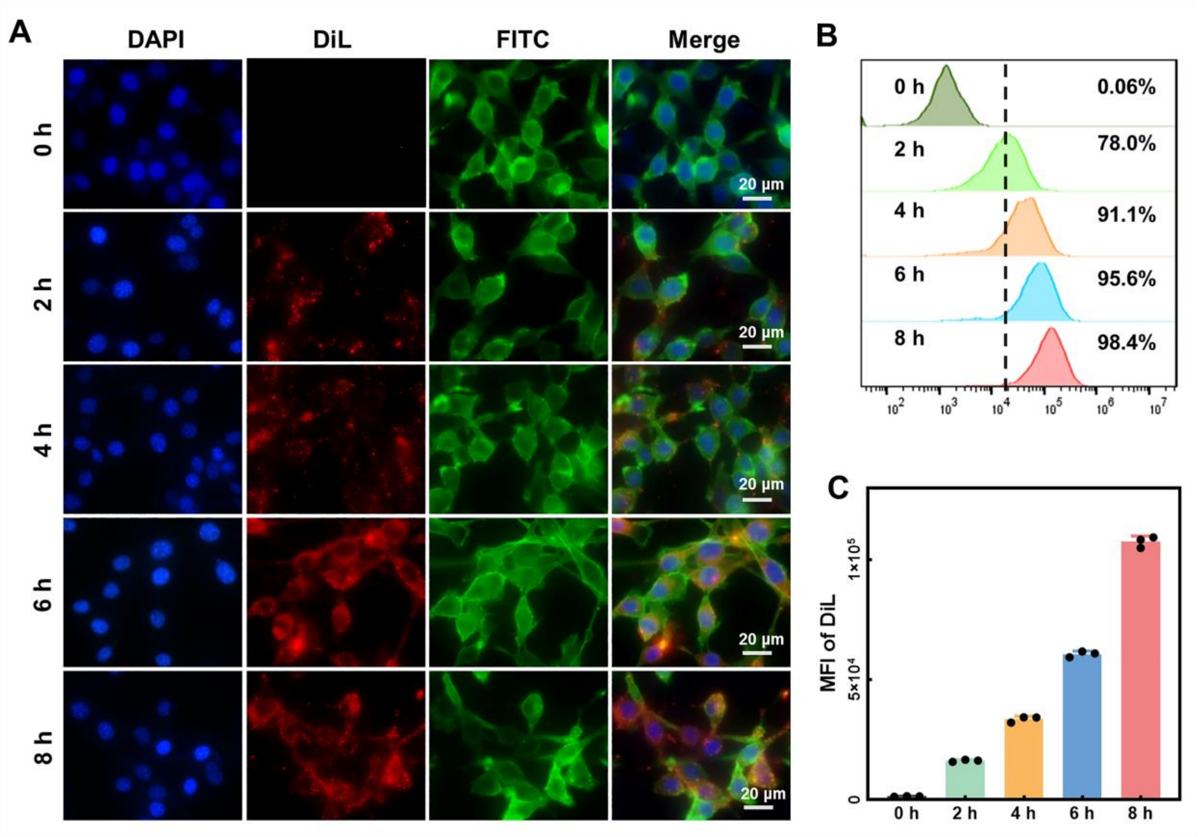

Figure S3. **Uptake of DiL@LM by CT26 cells after different incubation times.** (A) Fluorescence images of CT26 cells after 0 h, 2 h, 4 h, and 8 h of co-incubation with DiL@LM. (B) Flow cytometric quantification of DiL$^+$ CT26 cells after incubation with DiL@LM for different durations. (C) Mean fluorescence intensity of CT26 cells after incubation with DiL@LM for different times. (n = 3). Data are presented as mean ± SD

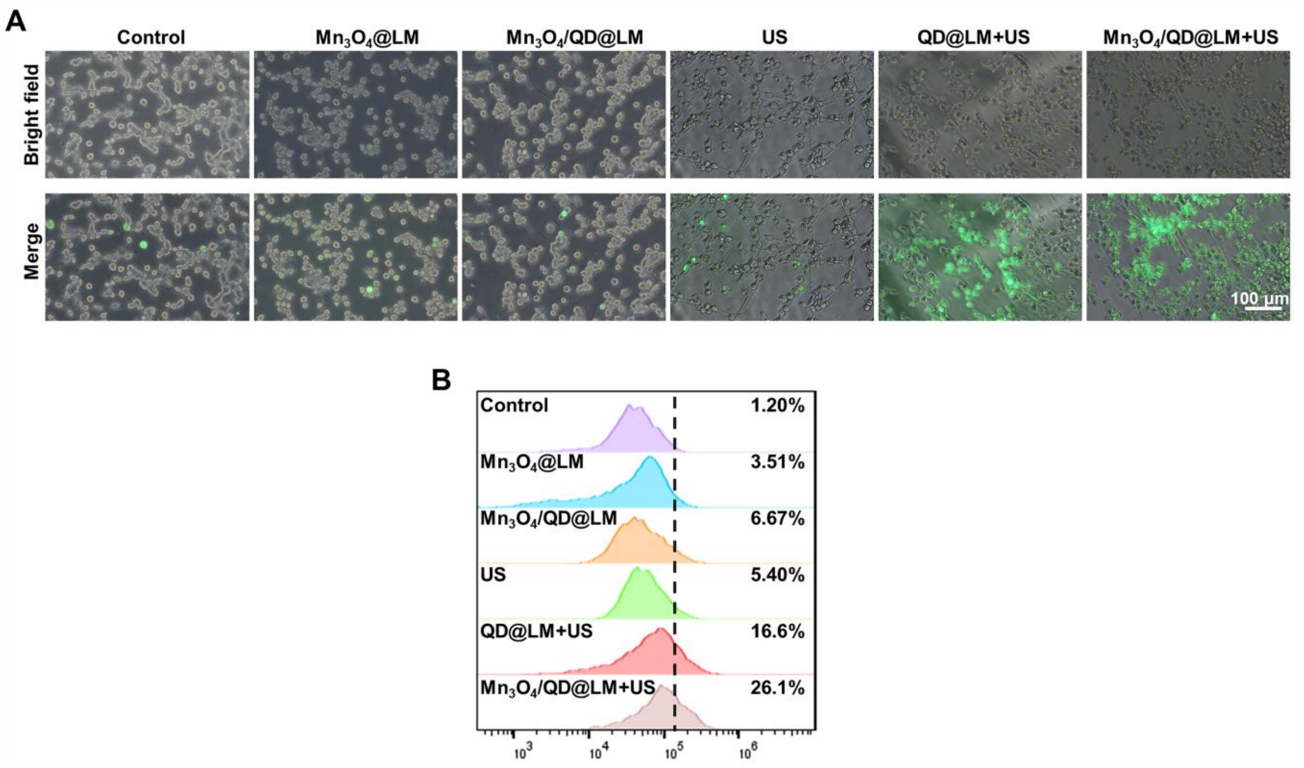

Figure S4. (A) Brightfield and fluorescence merge images of DCFH-DA-stained CT26 cells following different treatments. (B) Flow cytometric analysis of ROS levels in CT26 cells after different treatments.

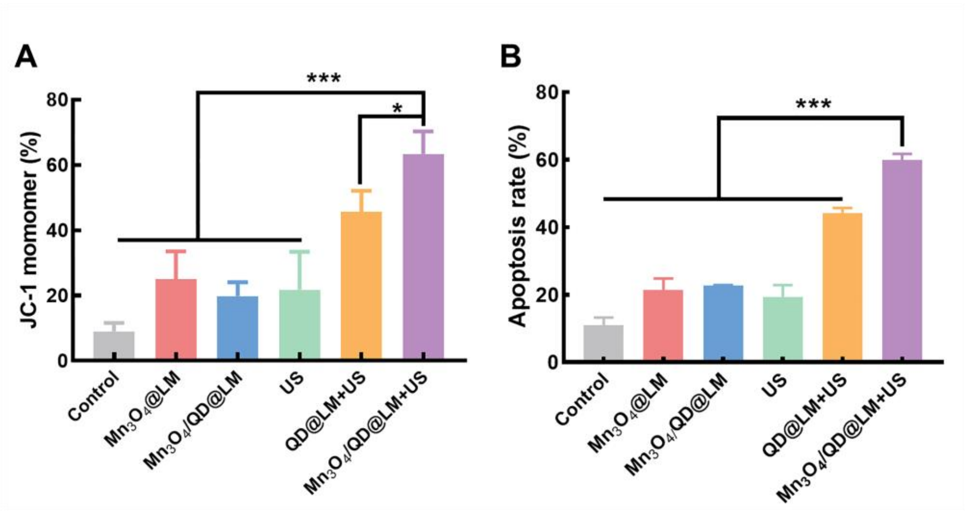

Figure S5. **The relative quantification results of MMP assay, and apoptosis assay.** (A) Quantitative analysis of MMP in CT26 cells measured by flow cytometry. (n = 3) (B) Quantitative analysis of apoptosis in CT26 cells measured by flow cytometry. (n = 3) Data are presented as mean ± SD. Statistical comparisons were performed by one-way ANOVA. Statistically significant difference: $*p < 0.05$, $**p < 0.01$, and $***p < 0.001$.

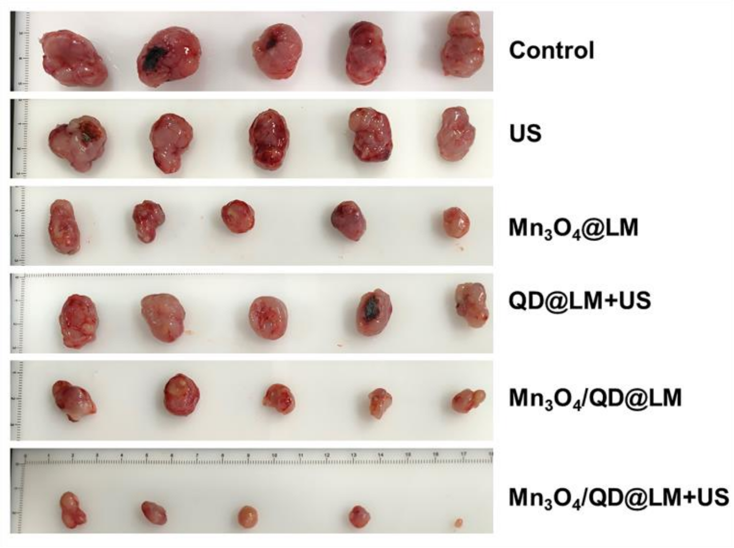

Figure S6. Photograph of excised tumors from mice in different treatment groups.

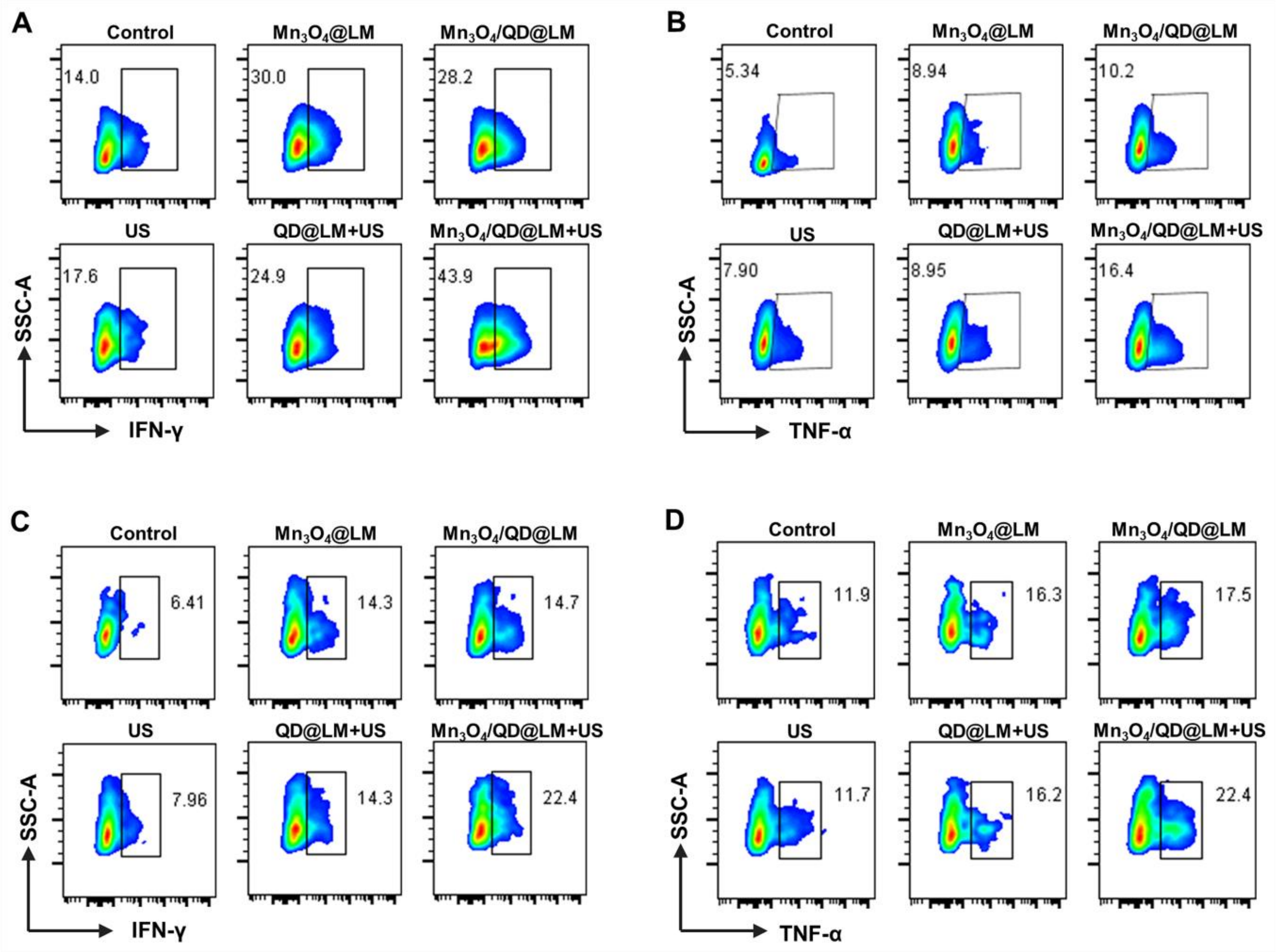

Figure S7. Cytokine levels in tumor tissues of mice in different treatment groups (A) Representative flow cytometry scatter plots of $CD8^+$ IFN-γ^+ T cells in tumor tissues from different treatment groups. (B) Representative flow cytometry scatter plots of $CD8^+$TNF-α^+ T cells in tumor tissues from different treatment groups. (C) Representative flow cytometry scatter plots of $CD4^+$ IFN-γ^+ T cells in tumor tissues from different treatment groups. (D) Representative flow cytometry scatter plots of $CD4^+$TNF-α^+ T cells in tumor tissues from different treatment groups.

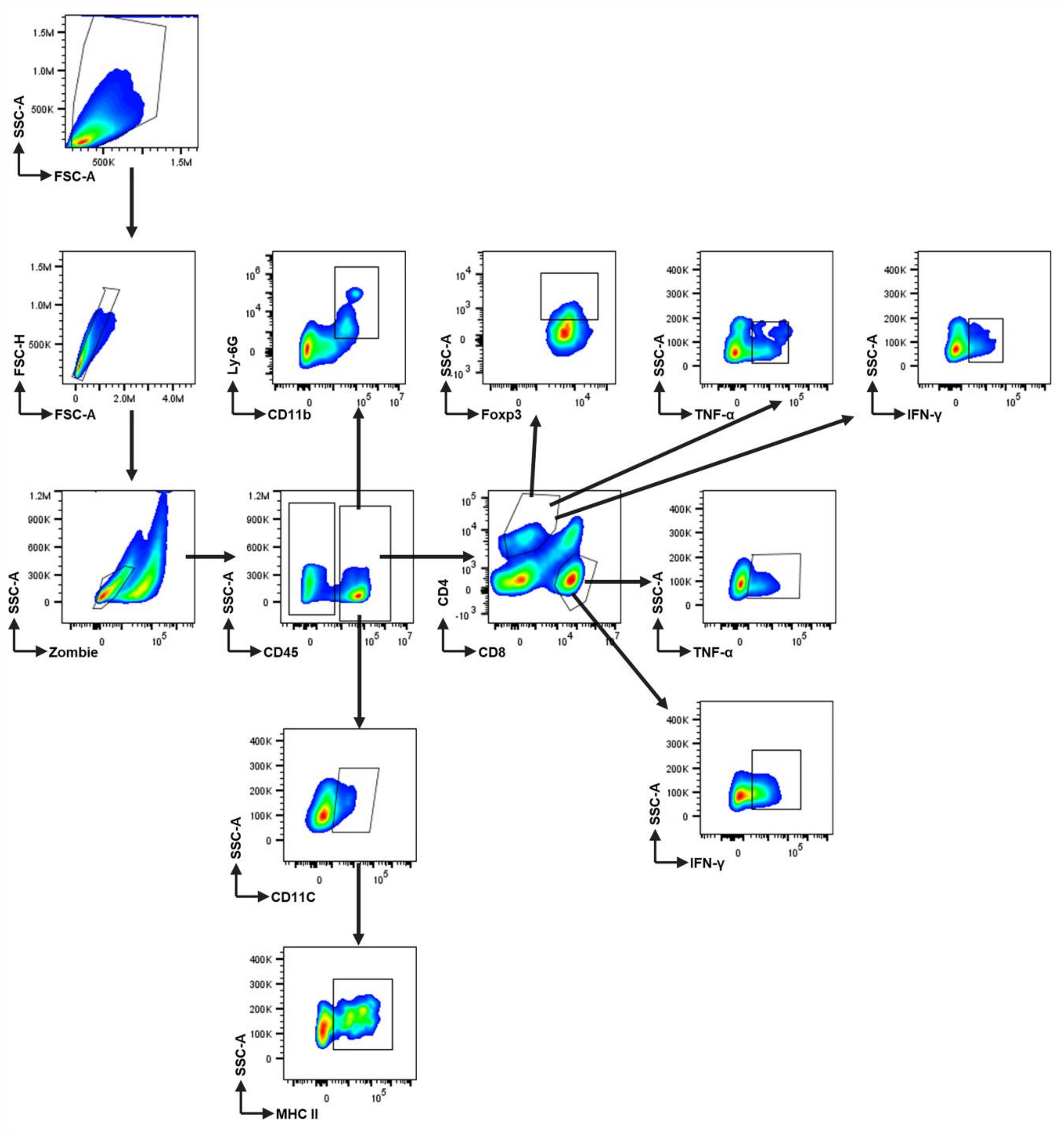

Figure S8. Gating strategy for flow cytometric analysis of mouse tumor tissues.
